# Research advances in the therapy of metabolic syndrome

**DOI:** 10.3389/fphar.2024.1364881

**Published:** 2024-07-30

**Authors:** Zitian Lin, Luning Sun

**Affiliations:** ^1^ Edinburgh Medical School, College of Medicine and Veterinary Medicine, The University of Edinburgh, Edinburgh, United Kingdom; ^2^ Zhejiang University-University of Edinburgh Institute, International Campus, Zhejiang University, Haining, China; ^3^ Department of Pathophysiology, College of Basic Medical Science, China Medical University, Shenyang, China

**Keywords:** metabolic syndrome, pathogenesis, therapy, statins, metformin, butyrate, probiotics

## Abstract

Metabolic syndrome refers to the pathological state of metabolic disorder of protein, fat, carbohydrate, and other substances in the human body. It is a syndrome composed of a group of complex metabolic disorders, whose pathogenesis includes multiple genetic and acquired entities falling under the category of insulin resistance and chronic low-grade inflammationand. It is a risk factor for increased prevalence and mortality from diabetes and cardiovascular disease. Cardiovascular diseases are the predominant cause of morbidity and mortality globally, thus it is imperative to investigate the impact of metabolic syndrome on alleviating this substantial disease burden. Despite the increasing number of scientists dedicating themselves to researching metabolic syndrome in recent decades, numerous aspects of this condition remain incompletely understood, leaving many questions unanswered. In this review, we present an epidemiological analysis of MetS, explore both traditional and novel pathogenesis, examine the pathophysiological repercussions of metabolic syndrome, summarize research advances, and elucidate the mechanisms underlying corresponding treatment approaches.

## 1 Introduction

Metabolic syndrome (MetS), also known as Reaven syndrome, syndrome X, and insulin resistance syndrome, is a significant global health issue. WHO defines it as a metabolic abnormality associated with visceral adiposity. It includes glycolipid metabolism characterized by typically systemic hypertension, central obesity, atherogenic dyslipidemia (including high-density lipoprotein cholesterol [HDL-c], low-density lipoprotein cholesterol [LDL-C], and hypertriglyceridemia), and insulin resistance. Although the diagnostic criteria for MetS have not been fully standardized globally, various international organizations use the following five indicators for assessment and diagnosis of MetS: central obesity (represented by waist circumference [WC] and body mass index [BMI], and waist/hip ratio), fasting glucose levels, triglyceride levels, HDL-c levels, and blood pressure. Table 1 lists five diagnostic criteria from the World Health Organization (WHO, 1998), the European group for study of insulin resistance (EGIR, 1999), the National Cholesterol Education Program Adult Treatment Panel III (ATPIII, 2001), the International Diabetes Federation (IDF, 2005), and the American Heart Association/National Heart, Lung, and Blood Institute (AHA/NHLBI, 2009). The prevalence of MetS in adults worldwide reaches more than 20%, and the incidence is increasing over time (Garralda-Del-Villar et al., 2018). The incidence of MetS is positively associated with the incidence of obesity and type 2 diabetes mellitus (T2DM), and approximately 85% of people with T2DM also have MetS (Alberti et al., 2009). People with MetS are 5 times more likely to develop T2DM than healthy people (Stern et al., 2004). At the same time, MetS increases the incidence of cardiovascular disease (CVD), and people with MetS have a 3 times greater incidence of heart attack than those without MetS (Garralda-Del-Villar et al., 2018). In addition, the incidence of MetS is also strongly associated with gender, eating habits, and sleep. In the Asia-Pacific region, the prevalence rate of MetS is higher in females than in males, which is related to hormonal homeostasis in females, specifically manifested in the increase of abdominal obesity and the decrease of HDL-C level (Pradhan, 2014; Ranasinghe et al., 2017). The control of MetS is also related to dietary patterns. Healthier eating patterns are linked to a lower incidence of MetS, while unhealthy eating patterns are related to a higher risk of MetS (Rodriguez-Monforte et al., 2017). In addition to differences in diet and sex, studies have found that sleep also seems to contribute to the occurrence of MetS, and skipping breakfast and sleeping too long or too short both increase the prevalence of MetS (Pan Y. et al., 2020). The presence of MetS has been related to many noncommunicable chronic diseases, such as T2DM and CVD. Since CVD is currently the leading cause of morbidity and mortality worldwide, and T2DM also endangers human health, it is crucial to investigate the treatment of MetS.

**TABLE 1:** Diagnosis criteria of metabolic syndrome.

Clinical measure	Criteria					Diagnosis	References
	Central obesity	Blood glucose	Triglyceride level	HDL-c level	Blood pressure		
WHO (1998)	• BMI > 30 kg/m^2^ and/or • Waist/hip ratio > 0.9 (men) or > 0.85 (women)	• Impaired fasting glucose or • Impaired fasting glucose or • T2DM diagnosis	• ≥150 mg/dL	• <35 mg/dL (men) or <39 mg/dL (women)	• <35 mg/dL (men) or <39 mg/dL (women)	≥3 criteria, one of which should be IR^a^	Reaven (1997)
EGIR (1999)	• WC > 37” (men) or >32” (women)	• Impaired fasting glucose or • Impaired glucose tolerance		• <39 mg/dL (men and women)	• <39 mg/dL (men and women)	≥3 criteria, one of which should be IR^b^	Balkau and Charles (1999)
ATPIII (2001)	• WC > 40” (men) or >35” (women)	• Impaired fasting glucose or • High blood glucose treatment or • T2DM diagnosis		• <40 mg/dL (men) or <50 mg/dL (women)	• <40 mg/dL (men) or <50 mg/dL (women)	≥3 criteria	Grundy et al. (2004)
IDF (2005)	• BMI > 30 kg/m^2^ • WC > 37” (men) or >32” (women) or		• ≥150 mg/dL or • Triglyceride treatment	• <40 mg/dL (men) or <50 mg/dL (women) or • HDL treatment	• ≥130 mmHg systolic and/or • ≥85 mmHg diastolic or • Hypertension treatment	≥3 criteria, one of which should be central obesity	Eckel et al. (2010)
AHA/NHLBI (2009)	• WC > 40” (men) or >35” (women)					≥3 criteria	Saklayen (2018)

Note. Impaired fasting glucose was defined as ≥110 mg/dL in 2001, but this was modified in 2004 to be ≥100 mg/dL. Impaired glucose tolerance is defined as 2 h glucose >140 mg/dL.

^a^ WHO IR, is defined as the presence of IR, impaired fasting glucose, or impaired glucose tolerance.

^b^ EGIR IR, is defined as plasma insulin levels >75th percentile.

## 2 Pathogenesis of metabolic syndrome

### 2.1 Insulin resistance exacerbates MetS by mediating free fatty acids

Insulin resistance (IR) means that the physiological effect of insulin in the body is weakened, the target cells are not sensitive to insulin, and relatively more insulin is required to maintain a normal blood glucose level. It is a common pathological mechanism of various metabolic-related diseases, especially T2DM (insulin-independent diabetes). The causes of IR may be insulin degradation, insulin structure abnormalities, insulin receptor defects, and insulin receptor binding abnormalities.

IR is closely associated with the occurrence and development of MetS. The primary manifestation of IR is the decrease of the body’s sensitivity to insulin, which mediates the production of free fatty acids (FFAs) by reducing glucose absorption by muscles and other tissues and destroying the anti-lipidysis effect of insulin. The increase of circulating FFAs, in turn, leads to the change of insulin signaling cascade in different organs, thus worsening IR (Girousse et al., 2013). FFAs, released in large quantities from the expanded fat tissue mass, lead to increased hepatic production of triglycerides (TGs), very low-density lipoprotein (VLDL), and glucose. It further leads to an increase in the synthesis of cholesterol esters and TGs, which promote the production of low-density lipoproteins (LDLs). These, in turn, activate cholesterol ester transfer protein (CETP), and it promotes the conversion of TGs from LDLs to high-density lipoprotein (HDL), thus increasing the HDL clearance rate and reducing the HDL concentration (Murakami et al., 1995). IR causes an imbalance in the concentration of these lipoproteins, leading to higher serum viscosity and thrombosis, causing atherosclerotic dyslipidemia and increasing the risk of CVD (Juhan-Vague et al., 2003).

In addition, FFAs suppress insulin-mediated glucose uptake in muscle, resulting in reduced glycogen synthesis and insulin sensitivity. This is because FFAs disrupt the insulin receptor substrate (IRS-1)-associated phosphatidylinositol 3-kinase (PI3K) activity, resulting in reduced translocation of glucose transporter type 4 (GLUT-4) to the cell surface, thereby decreasing muscle glucose uptake (Griffin et al., 1999). Free FFAs act on the liver, promoting lipogenesis and gluconeogenesis, which increases insulin secretion from the pancreas to a certain extent, leading to hyperinsulinemia. Eventually, however, this insulin compensation fails, resulting in reduced insulin secretion, and the lipotoxic effects of FFAs on pancreatic beta cells further reduce insulin levels (Eckel et al., 2005). At the same time, hyperinsulinemia leads to enhanced sodium reabsorption by increasing sympathetic nervous system (SNS) activity, further promoting the development of hypertension and the risk of T2DM. Therefore, excess circulating FFAs are significant factors leading to the development of IR. Because IR leads to reduced insulin secretion, insulin-mediated vasodilation is weakened. Moreover, FAAs increase soluble endothelial activity markers, reduce the production of vasoprotective molecule nitric oxide (NO) in vascular endothelial cells, and increase oxygen species (ROS), leading to FFAs-induced oxidation of LDL, vasoconstriction, and expression of lipoprotein receptor-1 (LOX-1) on endothelial cells (Ghosh et al., 2017b). FFAs can cause oxidative stress that promotes the development of IR. Oxidative stress refers to the imbalance of REDOX reaction in the body and the sharp increase of ROS in the body, thus destroying the structure and function of lipids and other biomolecules and ultimately leading to the generation of obesity, MetS, diabetes, and other chronic metabolic diseases (Conde et al., 2010b). ROS aggravates MetS through lipid peroxidation: ROS can induce the oxidation of polyunsaturated fatty acids on biofilms through iron-dependence or lipoxysynthase catalysis, destroy the double-layer arrangement of membrane lipids, and inactivate membrane binding receptors and enzymes to increase tissue permeability (Mohamedali et al., 1998). The NO decrease can lead to endothelial dysfunction caused by FFA-induced vasoconstriction and loss of insulin’s vasodilatory effect (Tripathy et al., 2003; Trayhurn and Wood, 2004).

**FIGURE 1:**
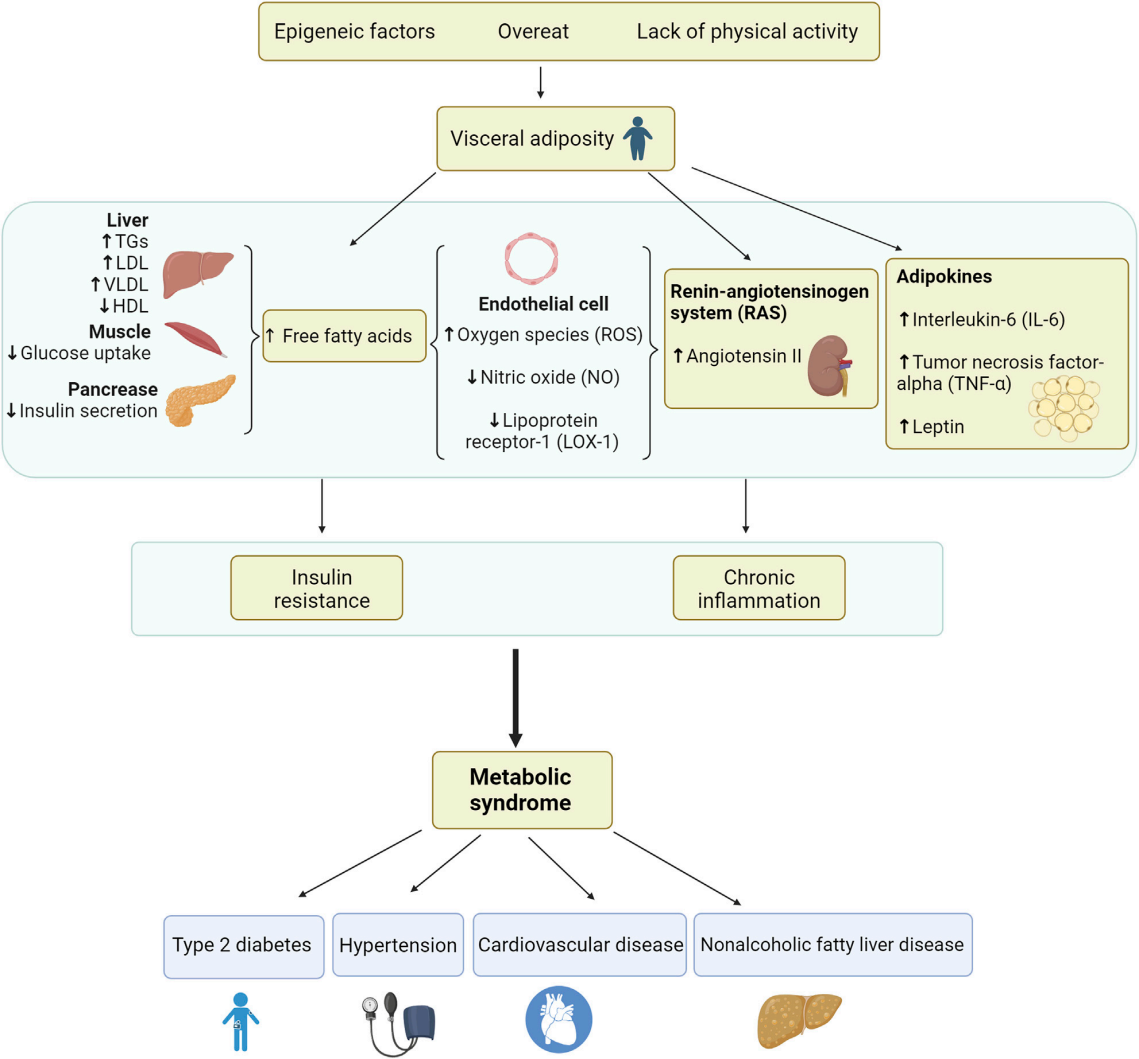
Pathogenesis of metabolic syndrome.

Moreover, excess circulating FFAs produce more interleukin-6 (IL-6) and tumor necrosis factor-alpha (TNF-α) through endocrine and paracrine effects of the pro-inflammatory state, promotes triglyceride lipolysis in adipose tissue, increases circulating FFAs promotion and the development of IR. Simultaneously, FFAs and cytokines such as IL-6 increase the production of hepatic fibrinogen and plasminogen activator inhibitor-1 (PAI-1), which supplements the PAI-1 overproduction in adipose tissue, leading to a thrombotic state (Eckel et al., 2005). The pathophysiology of insulin metabolic syndrome is shown in Figure 1.

### 2.2 Adipose tissue promotes MetS by secreting proinflammatory factors

The recent discovery of the endocrine function of adipose tissue provides more mechanistic explanations for the occurrence of MetS (Ghosh et al., 2017a). Some adipokines released by adipose tissue play a prominent role in the pathophysiology of MetS, including peptides (e.g., plasminogen activator inhibitor [PAI-1], angiotensin-converting enzyme), hormones (e.g., leptin) and inflammatory cytokines (e.g., IL-6, TNF-α, monocyte chemotactic protein [MCP-1] and chemerin) (Bernberg et al., 2012).

IL-6 is a potent inflammatory cytokine released by macrophages and adipocytes. IL-6 levels are elevated in adipose tissue of diabetic and obese patients, especially those with MetS characteristics, and elevated IL-6 concentrations have been reported to be related to hypertension, atherosclerosis, and cardiovascular events (Hotamisligil et al., 1994; Kim et al., 2012). This is because IL-6 mediates insulin resistance by regulating fat and glucose metabolism (Kanemaki et al., 2010). TNF-α is another cytokine released mainly by local macrophages in adipose tissue. It is an essential regulator of many cardiovascular pathologic conditions and correlates with insulin resistance. TNF-α disrupts insulin signaling in adipocytes and hepatocytes by phosphorylating serine and inactivating the insulin receptor and downstream signaling molecules, thus playing its pathogenic role and weakening insulin metabolism (Considine et al., 1996). TNF-α mediates the induction of lipolysis in the liver, thereby promoting insulin resistance and increasing circulating FFA levels (Kahn et al., 2019).

Leptin has been verified to be positively associated with body fat levels and obesity (Obradovic et al., 2021). It promotes energy expenditure and inhibits food absorption while maintaining glucose homeostasis and controlling insulin sensitivity (Pérez-Pérez et al., 2020). However, the metabolic imbalance in obesity cannot be corrected by high leptin, which results in the tissue of obese patients becoming less sensitive to leptin, triggering “leptin resistance” (Conde et al., 2010a). In addition, leptin has a regulatory effect on various immune cells, promoting the maturation and differentiation of innate immune cells, producing pro-inflammatory cytokines TNF-α, and mediating chemotaxis of inflammatory infiltration. At the same time, leptin also regulates adaptive immune cells and promotes the transformation of regulatory T cells into Th1 cells, thus producing more pro-inflammatory factors (Patel et al., 2008; Birben et al., 2012). Because higher leptin levels are associated with increased inflammation and cardiovascular risk, leptin is considered a primary factor in MetS, obesity, and cardiovascular disease (Su et al., 2019).

In addition, the renin-angiotensinogen system (RAS) is also involved in the development of MetS. During MetS development, adipose tissue releases an angiotensin-converting enzyme, which promotes the production of the peptide angiotensin II, which produces ROS by activating the enzyme nicotinamide adenine dinucleotide phosphate (NADPH) oxidase (Saiki et al., 2009). Together, ROS, LOX-1, and RAS form a vicious cycle that induces fibroblast proliferation, endothelial dysfunction, and inflammation, leading to dyslipidemia and an increased risk of atherosclerosis and hypertension in MetS.

### 2.3 Novel studies about the pathogenesis of metabolic syndrome

Fetuin-A, also known as α2-hermans-schmid glycoprotein (AHSG), is a protein secreted by the liver with pleiotropic metabolic effects. Studies have shown that the level of circulating fetuin-A in MetS patients is higher than in patients without MetS, which may be a tendency to increase the risk of MetS with the increase of circulating fetuin-A concentration (Pan X. et al., 2020). One of the ways that Fetuin-A promotes MetS is that Fetuin-A promotes insulin resistance (Srinivas et al., 1993). The mechanism of fetuin-A mediating insulin resistance is through inhibiting the phosphorylation of IRS-1 and downstream molecules of the PI3k/Akt pathway and the tyrosine kinase activity of the insulin receptor (Fahed et al., 2022). Fetuin-A knockout mice were found to have impaired insulin resistance, as indicated by increased glucose clearance, enhanced insulin sensitivity, and reduced serum FFAs and TGs (Fahed et al., 2022). In addition, fetuin-A mediates the migration and infiltration of macrophages into adipose tissue through chemical attractants, inducing the release of inflammatory factors, which in turn promotes the development of MetS (Siegel-Axel et al., 2014).

In addition to being the primary energy source generated by aerobic respiration, mitochondria are also the primary source of ROS production because, in the electron transport chain (ETC), a small number of high-energy electrons are leaked and react directly with oxygen (Brookes, 2005). However, there are mechanisms in mitochondria to maintain the balance of ROS by activating enzymes such as superoxide dismutase to remove ROS produced by themselves or other organelles such as peroxisomes (Andreyev et al., 2005; Wang B. et al., 2013). Thus, mitochondrial dysfunction can cause impairment of mitochondria antioxidant mechanism, resulting in overproduction of ROS, causing cell damage and “oxidative stress” that promotes the development of many diseases, including MetS (Fahed et al., 2022).

## 3 The pathophysiological repercussions of metabolic syndrome

Metabolic abnormalities associated with visceral adiposity, including dyslipidemia, hypertension, IR, and central obesity, have been found in patients with MetS. Hence, MetS patients suffer damage to the pancreas, vascular system, and liver tissue caused by high blood pressure, abnormal blood glucose, dyslipidemia, and inflammation (Grundy, 2008; Tariq et al., 2016). These damages include diabetic cardiomyopathy, metaflammation, myocardial infarction (inflammation-mediated damage to the heart), cardiovascular-kidney-metabolic syndrome, and so on (Mastrocola et al., 2018; Crisafulli et al., 2020; Ndumele et al., 2023; Omoto et al., 2023).

### 3.1 Diabetic cardiomyopathy

Diabetic cardiomyopathy was first described in 1972 in diabetic patients with symptoms of heart failure (Rubler et al., 1972). Diabetic cardiomyopathy is a specific form of heart disease that is described as diabetes-induced heart failure in the absence of valvular disease, high blood pressure, and coronary artery disease (Dillmann, 2019). Due to a large amount of extracellular matrix (ECM) deposition, there is perivascular and interstitial fibrosis in the myocardium of diabetic cardiomyopathy, which leads to reduced myocardial diastolic compliance and hypertrophy and further causes heart failure. Insulin resistance, prevalent in patients with MetS, is a strong predictor of T2D, contributing in part to this metabolic form of cardiomyopathy (Bugger and Abel, 2014). Studies on the hearts of diabetic rodents and human models have shown that hyperglycemia and insulin resistance increase fatty acid uptake and oxidation in the heart (Peterson et al., 2004; Buchanan et al., 2005; Bugger et al., 2008). The increase in fatty acid uptake beyond the use of the heart promotes the deposition of ceramide and triacylglycerol, which leads to steatosis and cardiomyocyte hypertrophy (Bugger and Abel, 2014; Levelt et al., 2018). In addition, increased oxidation of fatty acids caused by high glucose levels leads to mitochondrial uncoupling to produce excess ROS. In addition, diabetes, as a proinflammatory state, increases the concentration of proinflammatory cytokines, activates Transforming Growth Factor beta (TGF-β)/Smad signal, and recruits and activates fibroblasts, further promoting ECM deposition (Diamant et al., 2005; Russo and Frangogiannis, 2016). ECM deposition and upregulation of TGF-β reduce ECM enzyme metalloproteinase activity, thus promoting the development of myocardial fibrosis (Westermann et al., 2007).

### 3.2 Metaflammation

Metaflammation is a chronic low-grade inflammatory state primarily arising from metabolic cells responding to excess energy and nutrients in metabolic tissues such as muscle, liver, adipose tissue, pancreas, and brain (Hotamisligil, 2006). It involves a repertoire of molecules and signaling pathways akin to classical inflammation (Hotamisligil, 2006). There is literature evidence that metaflammation is a significant connection between the development of metabolic disorders and elevated risk of CVD. Studies have found that oxidative stress, insulin resistance, T2DM, and obesity are strongly associated with chronic inflammation, which is characterized by abnormal cytokine production, activation of inflammatory signaling pathway networks, and increased reactants in the acute phase (Dandona et al., 2004; Wellen and Hotamisligil, 2005; Shoelson et al., 2006). Specifically, the whole MetS factors play a role in the formation of metaflammation, an inflammatory state linked to the activation of the early innate immune response via the assembly of the multiprotein complex inflammasome. The most studied is the NOD-like receptor pyiridin domain containing (NLRP) 3 inflammasome family. The NLRP3 inflammasome is a pattern recognition receptor (PRR) belonging to the NOD-like receptors (NLRs) family. The NLRP3 inflammasome was proposed to induce insulin resistance, obesity-induced inflammation, and heart diseases (Vandanmagsar et al., 2011; Collino et al., 2013; Toldo et al., 2016). The study found that in the case of diabetes and MetS, thioredoxin-interactingprotein (TXNIP) causes cardiac hypertrophy and myocardial injury by activating the NLRP3 inflammasome in cardiomyocytes (Liu et al., 2014; Higashikuni et al., 2023). In the ischemic heart, the NLRP3 inflammasome may be activated and induce caspase-1 activation to trigger inflammation and further cause cardiac dysfunction (Toldo et al., 2015). In MetS, uncontrolled activation of the transcription factor sterol regulatory element binding protein SREBP1c, which is involved in adipogenesis, leads to the accumulation of ceramides and palmitates (Wang et al., 2006). In hepatitis C-infected livers, the activation of SREBP and NLRP3 is mutually observed, and in the liver of STZ-induced diabetic rats, suppression of TNXIP causes concurrent downregulation of SREBP and NLRP3 activity (Wang W. et al., 2013; McRae et al., 2016). Therefore, a significant correlation exists between hepatic lipid metabolism imbalance and NLRP3 inflammasome activation.

### 3.3 Myocardial infarction

MetS is often accompanied by CVD, which leads to the production of extracellular vesicles (EVs) biased towards pro-inflammatory phenotypes. EVs are a group of nanoscale vesicles of different sizes, loads, and surface markers. EVs are released as membrane-bound vesicles, which help in various physiological and pathological processes by facilitating interorgan communication (Liu et al., 2021). EVs also play an essential role in maintaining the interaction between the structure and function of the heart. EVs facilitate intercellular communication within the cardiovascular system, regulating physiological tissue function and propagating detrimental signals during cardiovascular disease pathogenesis. The study found that circulating levels of EVs were elevated in both myocardial infarction (MI) mice and people (Akbar et al., 2017) and that EVs also accumulated in the heart during the early stages of MI (Deddens et al., 2016; Khandagale et al., 2022). It has been found that extracellular vesicles derived from M2 macrophages carrying circUbe3a facilitate the migration, proliferation, and phenotypic transformation of myocardial fibroblasts and contribute to fibrosis following acute MI (Wang et al., 2021). Consequently, EVs hold immense potential for both monitoring and therapeutic interventions in the context of cardiovascular disorders. It was found that phagocytic monocytes engulfed extracellular vesicles (EVs) derived from necrotic cardiomyocytes, facilitating the secretion of cytokines such as IL-6, CCL (chemokine ligand) 7, and CCL 2 (Loyer et al., 2018). Endothelial EVs in diabetic mice impede the process of angiogenesis and vascular remodeling following myocardial infarction and skeletal muscle ischemia (Cheng et al., 2021; Huang et al., 2022). Moreover, EVs-mediated intercellular communication between immune cells and fibroblasts has been implicated in the pathogenesis of cardiac fibrosis. Previous studies have demonstrated that exposure of macrophages to a hyperglycemic environment, mimicking diabetic conditions, leads to the release of EVs containing human antigen R, which subsequently stimulate fibroblasts to upregulate collagen synthesis, thereby promoting cardiac fibrosis (Govindappa et al., 2020).

### 3.4 Cardiovascular-kidney-metabolic syndrome

Cardiovascular-kidney-metabolic (CKM) syndrome is a complex disorder made up of CVD, T2DM, obesity, and kidney disease, so people with MetS often have CKM syndrome as well. Over time, the diseases in CKM syndrome progressively converge, leading to the development of subclinical coronary atherosclerosis (manifested by coronary artery calcification) and subclinical abnormalities in myocardial structure and function. Additionally, there is a gradual decline in kidney function, placing individuals at high risk for clinical cardiovascular disease, kidney failure, disability, and mortality. CKM syndrome is divided into four stages: Stage 0, the prevention and protection stage, where there are no risk factors for CKM syndrome (normal blood glucose, normal blood pressure, normal lipids, normal weight, normal kidney function). Stage 1 is excessive obesity (abdominal obesity and overweight) and adipose tissue dysfunction (high blood sugar and reduced glucose tolerance). Stage 2, moderate to high-risk stage of CKM syndrome, with hypertension, high triglycerides, T2DM, or MetS. Stage 3, the very high-risk stage of CKD syndrome, predicts the high-risk stage of CVD, and CKM overlaps with subclinical CVD risk factors (adipose tissue dysfunction or MetS). Stage 4, CKM overlaps with clinical CVD (heart failure, coronary heart disease, etc.) risk factors, divided into stage 4a (without kidney failure) and stage 4b (with kidney failure) (Ndumele et al., 2023).

## 4 Treatments for metabolic syndrome

### 4.1 The classic drugs for metabolic syndrome

#### 4.1.1 Statins

Statins can antagonize the conversion of hydroxymethylglutaryl-coenzyme (HMG-CoA) to mevalonate via inhibiting HMG-CoA reductase, the rate-limiting enzyme in the cholesterol biosynthesis pathway. In the liver, statins reduce cholesterol synthesis, resulting in increased cell-surface LDL receptor expression and increased microsomal HMG-CoA reductase production. This helps to improve the clearance of LDL from the blood, thereby reducing the amount of LDL in the body and alleviating MetS.

Statins can relieve insulin resistance through lipid-lowering and non-lipid-lowering effects. The non-lipid-lowering effects of statins refer to the inhibition of isoprene production, a vital lipid adjunct involved in the post-translational modification of various proteins, such as small GTP-binding proteins, and interference with cell signaling. Statins can inhibit the activation of protein kinase C (PKC) by downregulating the expression of Rho protein, thus weakening the phosphorylation of insulin signaling proteins such as insulin receptor (InsR) and insulin receptor substrate (IRS). It increased the phosphorylation of tyrosine residues of InsR and IRS and alleviated IR. Statins also activate phosphoesterinositol-3-kinase (PI-3K) and promote the activation of phosphoinositol-dependent protein kinase (PDK1), which phosphorylates protein kinase B (PKB), improves the insulin sensitivity of the receptor and reduces IR. Statins can reduce the level of Caveolin-1, promote the activity of endothelial nitric oxide synthase (eNOS), increase the production of nitric oxide (NO) (Kinlay, 2005), dilate blood vessels, increase circulating blood volume, facilitate tissue transport and utilization of blood sugar and insulin, and indirectly reduce IR. Finally, statins can reduce the production of free fatty acids and ameliorate IR through a lipid-lowering effect. In MetS, islet β cells are prone to inflammatory reactions and oxidative stress damage. The increase in the concentration of inflammatory factors and oxidative stress products can induce Nuclear Factor kappa-light chain-enhancer of activated B cells (NF-κB) activation, TNF-α gene expression increase, and so on to trigger cell apoptosis. At the same time, ROS acts on the leukocyte chemokines and arachidonic acid in the blood to produce leukotrienes and prostaglandins, respectively. The chemokines infiltrate the leukocytes, promote the release of inflammatory factors, and aggravate the inflammatory response, which in turn improves the sensitivity of β cells to oxygen free radicals, further stimulating the inflammatory cells and beta cells to produce free radicals, forming a vicious cycle, causing progressive damage to β cells. By inhibiting the activation of NF-κB and inhibiting the expression of pro-inflammatory cytokines such as IL-1β, TNF-α and inducible nitric oxide synthase (iNOS), statins can play anti-inflammatory and antioxidant effects and reduce the damage of pancreatic β cells (Wagner et al., 2002).

There are difficulties in identifying and diagnosing statin toxicity, but currently, known statin-associated muscle symptoms (SAMS) are the most common adverse effects of statins. The prevalence of SAMS varies with different statins. Patients taking lipophilic statins, such as simvastatin and atorvastatin, are at the highest risk for SAMS because of their nonselective spread to extrahepatic tissues, such as skeletal muscle. In contrast, hydrophilic statins, such as pravastatin and fluvastatin, are less risky due to their lower muscle penetration. It has been reported that more than half of SAMS may be caused by the combination of statins and drugs metabolized by the same hepatic cytochrome P450 isoforms (Muntean et al., 2017). Other side effects of statin may be more severe, including new-onset type 2 diabetes mellitus, neurocognitive effects, neurological and renal toxicity, and hepatotoxicity.

#### 4.1.2 Metformin

**FIGURE 2:**
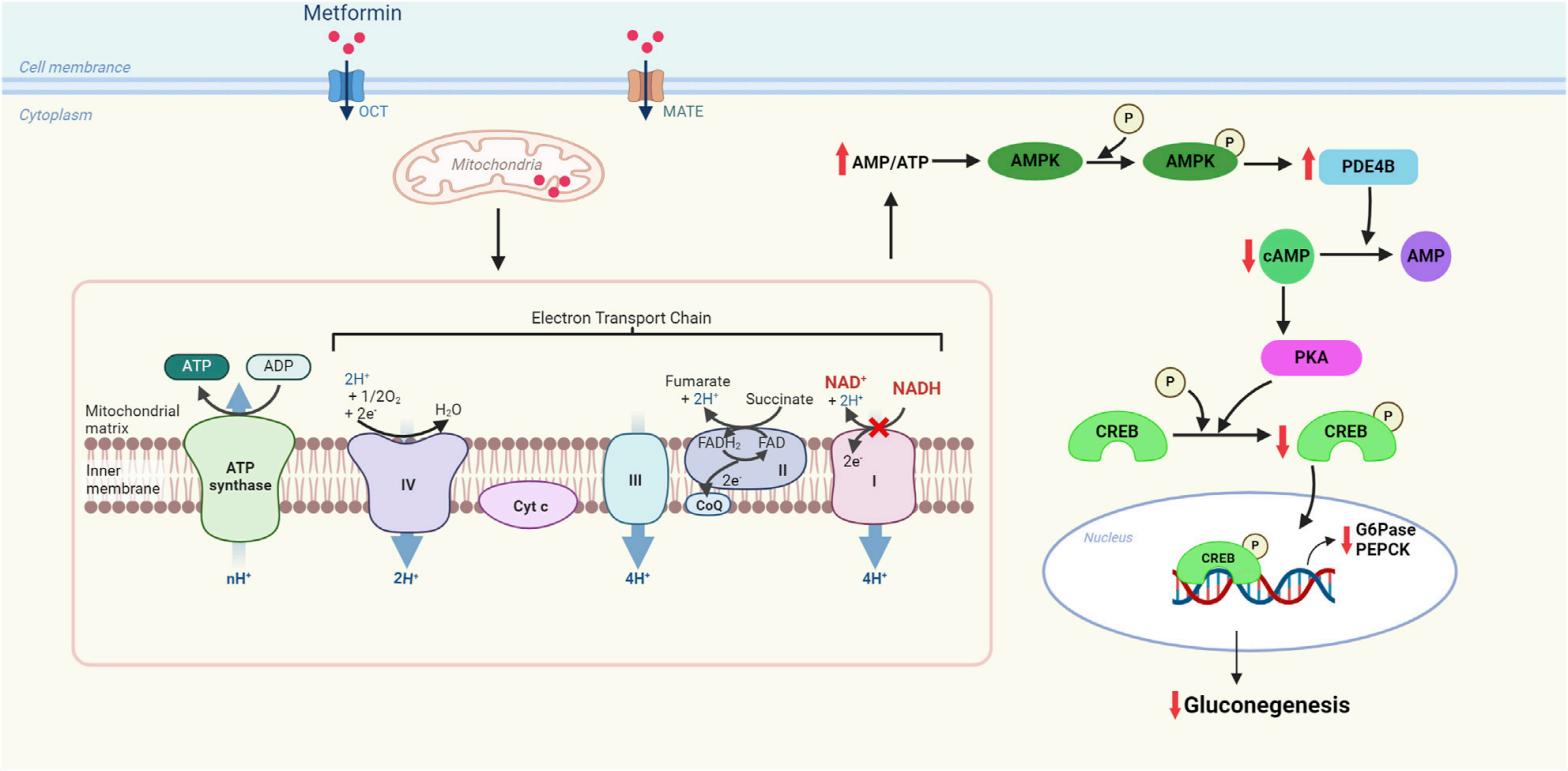
Molecular mechanism of metformin for inhibiting gluconeogenesis in the electron transport chain of mitochondria. Metformin has a brief and specific inhibitory effect on respiratory chain complex I (NADH-ubiquinone oxidoreductase), increasing the AMP/ATP ratio and restraining gluconeogenesis through the downregulation of the expression of gluconeogenic genes. OCT: organic cation transporter, a transporter that promotes cellular uptake of metformin; MATE: multidrug and toxin extrusion transporter, a carrier that carries metformin in and out of cells; AMPK: AMP-activated protein kinase; cAMP: cyclic AMP; PDE4B: phosphodiesterase 4B; PKA: protein kinase A; CREB: cAMP-response element-binding protein; G6Pase: glucose-6-phosphate; PEPCK: phosphoenolpyruvate carboxykinase.

Metformin is the most commonly used insulin-sensitizing agent in IR diseases such as diabetes, MetS, and obesity. The main effect of metformin in improving insulin resistance is by inhibiting the production of hepatic glucose. Metformin-mediated ETC complex I inhibition inhibits ATP production, resulting in elevated intracellular ADP and AMP levels and a consequent increase in the AMP/ATP ratio (Garcia and Shaw, 2017). The elevated AMP/ATP ratio activates AMP-activated protein kinase (AMPK), a primary bioenergy metabolism regulator in eukaryotic cells, which promotes catabolic reactions, produces more ATP, and inhibits anabolic pathways of energy consumption such as gluconeogenesis (Figure 2). Gluconeogenesis is the process by which simple non-sugar precursors (lactate, glycerol, glycogenic amino acids, etc.) are converted to glucose and other carbohydrates. It is regulated by the critical regulator of hepatic glucose output called the cAMP response element-binding (CREB) co-activator complex. CREB co-activator complex can regulate gluconeogenesis by regulating the activation of transcription of gluconeogenic genes, particularly glucose-6-phosphatase (G6Pase) and phosphoenolpyruvate carboxykinase (PEPCK). Activation of AMPK phosphorylates Ser436 of CREB-binding protein (CBP), evoking the disassembly of the CREB co-activator complex and then suppressing the expression of gluconeogenic genes (Shaw et al., 2005). Therefore, metformin affects the ability of liver cells to process glucose. Metformin can inhibit glucose production in the liver and increase glucose utilization rate in adipose tissue and skeletal muscle. This is because metformin is an insulin sensitizer that improves insulin sensitivity by affecting tyrosine kinase activity and insulin receptor expression. Significantly, unlike other hypoglycemic drugs, metformin does not usually stimulate endogenous insulin production and, therefore, does not put patients at risk for hypoglycemia (Panchapakesan and Pollock, 2018). Moreover, metformin has been shown to improve intestinal secretion of glucagon-like peptide 1 (GLP-1), a glucose-lowering gut incretin hormone that regulates glucose homeostasis and insulin secretion by inhibiting glucagon release from the pancreas (Bahne et al., 2016). Therefore, metformin can lower blood glucose, relieve IR, and reduce blood lipid, thus alleviating MetS.

In addition, the current study found that metformin’s treatment of IR is related to its effect on the expression of GLUT4 (Herman et al., 2022). GLUT4 is an insulin-regulated glucose transporter, and its level directly affects glucose utilization of target tissues because it mediates glucose transmembrane transport, a rate-limiting step in peripheral glucose disposal (Zisman et al., 2000a; Armoni et al., 2005). Impaired GLUT4-mediated glucose uptake is a significant mechanism that induces IR, which has been demonstrated in many studies. In a study of adipocytes in diabetic mice, glucose transport decreased by 45% after 5 days of diabetes compared to controls, and at 10 days, glucose transport was severely reduced, and insulin-stimulated reduced glucose transport was observed (Napoli et al., 1995). Data obtained from a study using transgenic specific knocking out GLUT4 in mouse muscle suggest that GLUT4 is involved in insulin-stimulated glucose transport in muscle and that specific knocking out GLUT4 led to IR in liver and adipose tissue (Zisman et al., 2000b). In addition, in studies of GLUT4 fat-specific knockouts in mice, it was found that GLUT4 protein expression was downregulated, leading to reduced insulin-stimulated glucose transport, causing insulin resistance and thus increasing the risk of diabetes (Abel et al., 2001). Studies have shown that no matter which tissues have a genetic defect in GLUT4, other insulin-target tissues are eventually affected (Graham and Kahn, 2007). Because metformin phosphorylates and activates AMPK, this may be related to its effect on GLUT4-mediated glucose transport. A study of adipocytes obtained from surgical biopsies and differentiated *in vitro* showed that after metformin treatment for 24 h, glucose uptake in human adipocytes increased by about 2.3-fold, and GLUT4 mRNA expression levels also increased (Grisouard et al., 2010). Some studies have found that metformin is vital in GLUT4 translocation by activating AMPK. Rab-GAP protein TBC1D1 is phosphorylated after AMPK activation, which controls the translocation of GLUT4 and plasma membrane levels (Hardie, 2013). The studies of 3T3-L1 preadipocyte cells and C2C12 skeletal muscle cell lines found that AMPK plays a crucial role in GLUT4 translocation (Lee et al., 2012; Hardie, 2013).

### 4.2 The novel drugs for metabolic syndrome

#### 4.2.1 Probiotics

Patients with MetS are often accompanied by abnormal blood glucose and dyslipidemia, which commonly lead to obesity. Probiotics can regulate blood glucose and lipids by promoting glucose metabolism and cholesterol conversion and inhibiting the absorption of glucose and lipids. It has been found that probiotic fermentation metabolites short chain fatty acids (SCFAs) (e.g., acetate, propionate, and butyrate) can act as signaling molecules to activate glucose-regulated protein 41 (GPR4l) and glucose-regulated protein 43 (GPR43) through free fatty acid receptors (FFARs) in the intestinal cells and then stimulate the secretion of peptide YY (PYY) and GLP-l, controlling energy intake and promoting intestinal gluconeogenesis to reduce appetite and enhance insulin sensitivity (Vernia et al., 2000; Krokowicz et al., 2014a). Moreover, the use of probiotics can improve gut microbes. *In vivo* studies have found that the SCFA metabolites can stimulate the proliferation and differentiation of colonic epithelium cells, increase mucus secretion, and prevent the destruction of tight linking proteins by reducing the uptake of lipopolysaccharides (LPS) on the cell wall of Gram-negative strains by intestinal cells, maintaining normal mucosal barrier permeability and protecting epithelial cells from exposure to harmful microbiota and pathogens that lead to intestinal microbiome disturbance (Topping and Clifton, 2001; Harte et al., 2011). The dysregulated microbiome contributes to pro-inflammatory reactions that are a hallmark of the obesity phenotype, which include increased uptake of LPS and regulation of intestinal barrier permeability, resulting in increased bacterial endotoxin in the circulation (Jaffar et al., 2016). Studies of human subjects found that systemic endotoxin levels were 20% higher in glucose-intolerant or obese people than in non-obese control (Harte et al., 2011). LPS and endotoxins can translocate to the circulation to promote cytokine production and initiate inflammation (Osborn and Olefsky, 2012). When LPS binds to toll-like receptors 4 and 2 (TLR 2, 4) in peripheral tissues and mucous membranes, macrophages and dendritic cells are activated, producing more inflammatory biomarkers (e.g., TNF-α and MCP-1) and initiating a cascade of pro-inflammatory signals. Studies in animals and humans have shown that probiotics play a vital role in modulating inflammation. After binding to specific receptors of intestinal epithelial cells, probiotics can inhibit regulatory T cells (Treg cells), NF-κB pathway, and pro-inflammatory cytokines produced by macrophages and neutrophils, thereby playing an anti-inflammatory role (Park et al., 2007; Vinolo et al., 2011).

**FIGURE 3:**
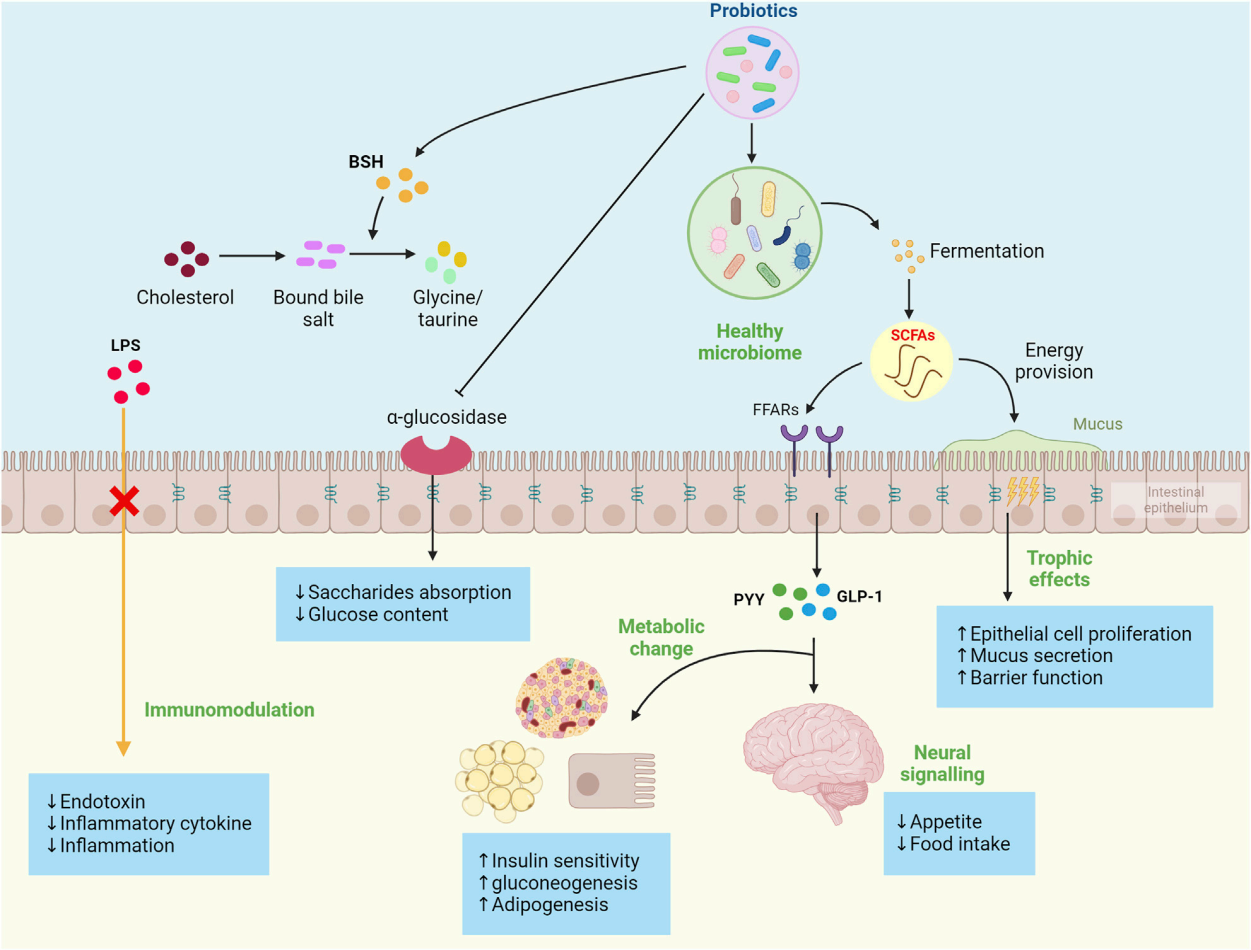
Summary of probiotics’ mechanism to improve metabolic syndrome development. These mechanisms include the production of probiotic fermentation metabolites short-chain fatty acids (SCFAs), regulation of cytokine production, reduced appetite through gut-brain signaling, enhanced adipogenesis and gluconeogenesis, inhibited the α-glucosidase, improve the activity of bile salt hydrolase (BSH), and ultimately increased insulin sensitivity and reduced proinflammatory factors.

Probiotics have also been shown to inhibit the activity of α-glucosidase in the intestine. Because the primary function of α-glucosidase is to directly participate in the hydrolysis of the glucosidic bond of starch and glycogen, releasing glucose as a product and increasing the content of glucose in the body, inhibiting α-glucosidase can delay the digestion and absorption of saccharides in the intestine. The main metabolic pathway of cholesterol in the body is to convert cholesterol into bile acid through the liver, and probiotics can not only improve the activity of bile salt hydrolase (BSH) but also contain a large amount of BSH. BSH can decompose bound bile saline into free bile salts and glycine/taurine, which promotes the *de novo* synthesis of bound bile salts from cholesterol to compensate for the loss, thereby reducing the serum cholesterol level in the body, thereby improving hypercholesterolemia. The mechanisms of probiotics are described in Figure 3. Table 2 summarizes the other effects of different probiotics on improving MetS.

**TABLE 2:** Animal models of probiotics effects in improving metabolic syndrome.

Probiotic strain	Studied model	Effect	Mechanism	References
*Lactobacillusfermentum* *Lactobacillus reuteri* FJSYC4-1 *and Lactobacillus reuteri* FGSZY33L6	Male C57BL/6J mice were treated with an HFD and 0.2 mL of 5 × 10^9^ CFU/mL bacterial solution for 11 weeks (n = 8)	↓ Food intake, weight gain, IL-6, INS, FBG, HOMA-IR, TC, LDL-C, LDL/HDL	Regulate gut microbiota and produces SCFAs	Zheng et al. (2021)
*Lactobacillus acidophilus* NX2-6	Male Kunming mice were fed with HFD and orally administered with 1 × 10^9^ CFU/mL of *L. acidophilus* NX2-6 (n = 10) for 8 weeks	↑ Glucose metabolism: adipose tissue (GK, p-Akt), liver (p-Akt, GK, GLUT2), small intestine (GK, GLUT2) ↓ FBG, INS, HOMA-IR, GLP-1	Treatment with *L. acidophilus* NX2-6 can promote glucose uptake, glycolysis, and intestinal gluconeogenesis, inhibit liver gluconeogenesis, and ameliorate HFD-induced glucose metabolism disorder by activating insulin signaling pathway	Tang et al. (2021)
*Lactobacillus plantarum* K8	The cell lysate of *L. plantarum* K8 was administered to 3T3-L1 preadipocytes every 2 days	↑ pAMPKα ↓ Lipid accumulation, PPARγ, C/EBPα, C/EBPβ, mRNA expression (DLK1, Krox20, and C/EBPδ), pJ AK2, pSTAT3/STAT5	Reduce lipid accumulation by regulating JAK/STAT and AMPKα signaling pathways	Kim et al. (2021)
*Bifidobacterium longum* BR-108	Male C57BL/6J mice were fed HFD for 5 weeks and were orally administered 200 mg/kg or 400 mg/kg BW of *B. longum* BR-108	↑ *Bifidobacterium* spp., *Lactobacillus* spp. ↓ Weight gain, liver weight, blood glucose, TC, insulin resistance, LPS	After sterilization, *B. longum* BR-108 can improve intestinal flora and prevent LPS inflow	Kikuchi et al. (2018)
*Akkermansia muciniphila* ATCC BAA-835	Male E3L.CETP mice were fed an 11% cholesterol and 0.05% cholate diet for 7 weeks and treated with 2 × 10^8^ CFU/200 μL of *A. muciniphila* ATCC BAA-835 for 4 weeks	↑ IL-10 (LPS stimulation) ↓ Body weight, TC, LPS, B cell, T cell, dendritic cell subpopulation activity, follicular B cells, mucosal B cells	Rapidly reduce hyperlipidemia and play an immunomodulatory role	Katiraei et al. (2019)
*Lactobacillus acidophilus*	Male C57BL/6 mice were supplemented with 3% DSS for 4 days. *L. acidophilus* (8 × 10^10^ CFU/kg) was administered 3 days after DSS administration	↑ Treg and IL-10 ↓ IL-17, TNF-a, IL-6 and IL-β	*L. acidophilus* can reduce inflammation by regulating the balance between Th17 and Treg cells	Park et al. (2018)

Note: HFD: high-fat diet, INS: insulin, FBG: fasting blood glucose, HOMA-IR: homeostasis model assessment of insulin resistance, TC: total cholesterol, LDL-C: low-density lipoprotein cholesterol, HDL: high-density lipoprotein cholesterol, GK: glucokinase, p-Akt: phosphorylated protein kinase B, GLUT2: glucose transporter 2, GLP-1: glucagon-like peptide 1, pAMPKα: phosphorylated-adenosine 5’- monophosphate-activated protein kinase α, PPARγ: peroxisome proliferator activated receptor γ, C/EBP- CCAAT, enhancer binding proteins, DLK1: delta-like 1 homologue, pJ AK2: Janus kinase 2, STAT: signal transducer and activator of transcription.

Data from several studies suggest that patients may improve current medical therapy by using probiotics in combination with other medications to treat MetS, but this will not affect related inflammatory biomarkers or clinical characteristics of the MetS (Aoki et al., 2017; Yoon et al., 2023).

#### 4.2.2 Butyrate

Butyrate, as a SCFA produced by gut microbes, can improve MetS. Multiple studies have found it can improve insulin sensitivity by stimulating adipogenesis (Nie et al., 2015). This is achieved through various mechanisms, mainly by increasing the expression of genes for adipocyte differentiation (He et al., 2009). For instance, the expression of SREBP-1c’s mRNA, which is a primary regulator for fatty acid *de novo* synthesis and adipogenesis, is activated by butyrate. In addition, butyrate promoted the upregulation of adipocyte differentiation markers C/EBPα, C/EBPβ, and PPARγ, promoting adipocyte differentiation and maturation. Increased adipocytes result in more lipids being broken down, and therefore, more lipogenesis diminishes fatty acids in the circulation, thereby protecting different organs from lipotoxicity and insulin resistance. Butyric acid can also reduce serum cholesterol levels.

Butyrate also acts on pancreatic islets to improve glucose homeostasis in patients with MetS. The study found a threefold increase in the number of islet cells but no longer produce any detectable islet hormones in T2DM patients compared to non-diabetic individuals. This dedifferentiation of islet cells is hypothesized to be caused by the pro-inflammatory cytokine IL-1β (Chen et al., 2019). IL-1β expresses upregulation in MetS and can induce pancreatic β-cell dysfunction and apoptosis (Francesca et al., 2016). The pancreatic β-cell is a type of cell whose function is reduced in patients with impaired glucose tolerance and T2DM. Butyrate can downregulate the expression of IL-1β-induced inflammatory genes to prevent IL-1β induced β-cell dysfunction and apoptosis. This is achieved by butyrate protecting β cells from IL-1β-induced impairment of insulin content and glucose-stimulated insulin secretion (GSIS) that characterize β cell dysfunction. Moreover, butyrate did not affect the expression of β cells-specific genes Ins, MafA, and Ucn3, which were reduced by IL-1β (Prause et al., 2021).

**FIGURE 4:**
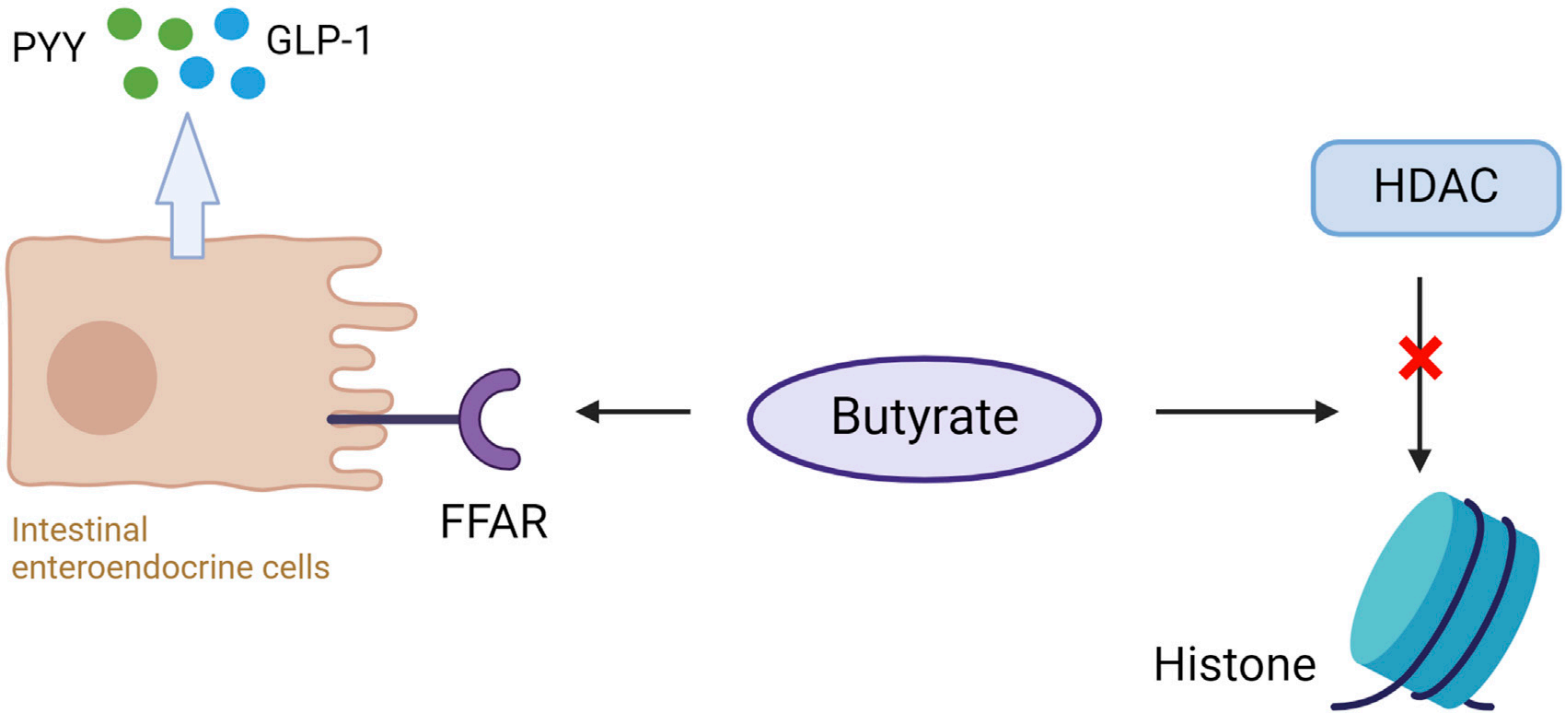
Butyrate binds to free fatty acid receptors (FFAR) on intestinal enteroendocrine cells to promote the release of peptide YY (PYY) and glucagon-like peptide (GLP-1). Butyrate simultaneously inhibits histone deacetylase (HDAC) activity, elevating histone acetylation (Ac) and gene expression modification.

**FIGURE 5:**
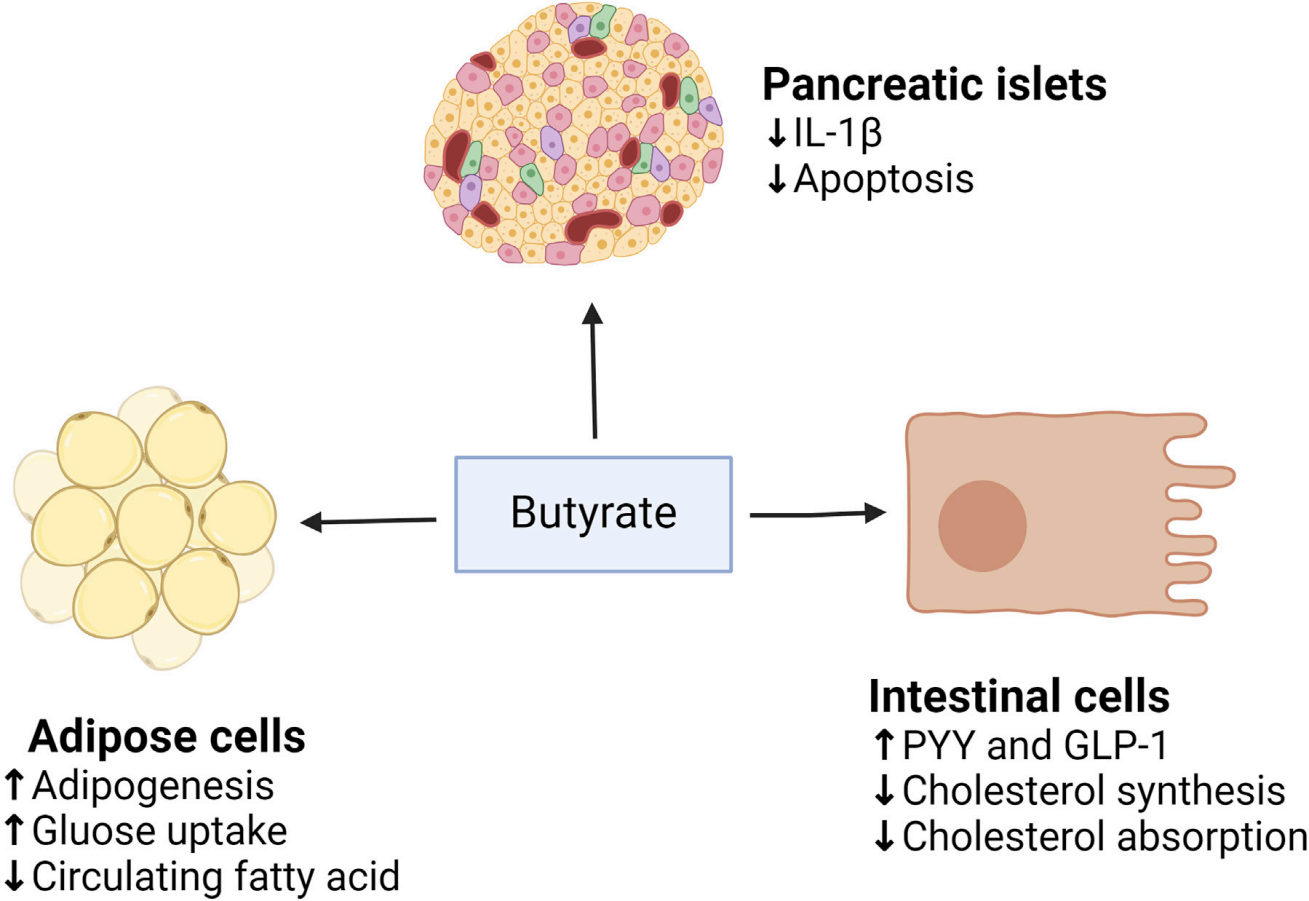
The roles of butyrate on adipose tissue, pancreatic islets, and intestinal cells. (↓), downregulation or inhibition; (↑) upregulation or activation.

Dietary supplementation with butyrate prevents or reduces obesity and insulin resistance (Chen et al., 2018a). This is because butyrate can bind to and activate the G protein-coupled FFARs, FFAR2, and FFAR3 in the intestinal enteroendocrine cells, leading to the release of PYY and GLP-1, which reduces appetite and body weight (Fahed et al., 2022). PYY slows gastric emptying and decreases appetite, whereas GLP-1 inhibits glucagon secretion and increases insulin secretion (Gao et al., 2009b), so butyrate can improve insulin sensitivity and alleviate MetS (Figure 4). The disorder of HDAC expression promotes the development of metabolic diseases such as diabetes and CVD (Yiew et al., 2015). Studies have shown that butyrate has an antidiabetic effect in T1D and T2DM animal models, related to the inhibition of histone deacetylases (HDACs) activation. HDAC inhibition and histone acetylation are related to β cell proliferation and differentiation and insulin gene regulation (Goicoa et al., 2006; Lenoir et al., 2011). In T1D mice models, after butyrate treatment, the body weight and the function of islet beta cells of diabetic mice were significantly improved, the blood glucose level was reduced considerably, and the plasma insulin level was increased, but insulin resistance was not restored (Khan and Jena, 2014). In high-fat diet (HFD) and streptozotocin (STZ) mediated T2DM mice models, butyrate treatment significantly reduced the increase of diabetes-related plasma triglyceride and cholesterol levels, reduced diabetes-related endocrine pancreatic (islet) damage and significantly inhibited the growth of diabetes-induced HDACs activity, preventing glucagon and forkhead box protein O1 (FOXO1)-mediated liver gluconeogenesis in type 2 diabetic rats (Khan and Jena, 2016). HDACs are a class of epigenetic modification enzymes that remove acetyl groups located in the tail of histones, preventing histone acetylation. Inhibition of HDACs will promote histone acetylation, which is conducive to the dissociation of DNA from histone octamer and relaxation of nucleosome structure so that various transcription factors and co-transcription factors can bind specifically to DNA binding sites and activate gene transcription (Bose et al., 2014). It also has been found that butyrate reduces the expression of the primary genes involved in intestinal cholesterol absorption, decreasing cholesterol absorption in the gut (Chen et al., 2018b). In addition, it decreases the activity of 3-hydroxy-3-methyl-glutaryl-coenzyme A (HMG-CoA) reductase (HMGCR) and then reduces cholesterol synthesis of Caco-2 enterocytes. The roles of butyrate in MetS are summarized in Figure 5.

In animal and human models, studies have found that butyrate treatment can reduce HFD-mediated weight gain and improve insulin sensitivity, hyperlipidemia, hyperinsulinemia, and hyperglycemia. Table 3 summarizes these studies. Currently, a lot of clinical trials also have focused on the effects of transrectal or oral administration of butyrate on intestinal disease, with several trials showing improvement in diverticulitis, travelers’ diarrhea, and ulcerative colitis (Krokowicz et al., 2014a; Steinert et al., 2017). No serious adverse effects occurred, and they were well tolerated. Oral butyrate, which causes foul-smelling belching, is a unique drawback (Krokowicz et al., 2014b). In the study on the effect of oral butyrate on MetS, it was observed that there was no increase in butyrate levels in plasma and feces after 4 weeks of oral butyrate treatment, and it did not improve glucose metabolism in MetS subjects. However, there are benefits for glucose metabolism in lean but non-MetS subjects (Kec et al., 2018). However, there are not enough human clinical trials investigating the benefits of butyrate on MetS.

**TABLE 3:** Experimental models of the effect of butyrate on indices of disorder in metabolic syndrome.

Models	Dose	Effect	References
HFD-fed male C57Bl/6 mice	100 mg/kg/day for 6 weeks	Reduced weight gain, fat BW %, total cholesterol, triglyceride, fasting glucose and insulin, AUC in GTT	Maria et al. (2017)
HFD fed male C57BL/6J mice	5 g/kg/day for 16 weeks	Increased energy expenditure and oxygen consumption Reduced weight, fat BW %, respiratory exchange ratio, fasting glucose and insulin, HOMA-IR, AUC in FBG	Gao et al. (2009a)
HFD fed APOE*3-Leiden.CETP mice	5% w/w in diet for 9 weeks	Reduced body weight, fat mass gain, triglyceride, plasma glucose, and insulin, HOMA–IR	Li et al. (2017)
HFD fed male C57BL/6JUnib mice	5% w/w in diet for 60 days	Reduced body weight, visceral fat accumulation, fasting glucose, fasting and fed insulin, AUC in ITT	Matheus et al. (2017)
HFD fed male C57BL/6J mice	400 mg/kg for 16 weeks	Reduced weight gain and serum insulin level Increased all-trans-retinal, α-linoleate, resolvin E1, p-AMPK, GLUT4 expression	Gao et al. (2019)
db/db mice	1.0 g/kg every other day for 6 weeks	Reduced fasting glucose, glucose in GTT and ITT	Wang et al. (2015b)
HFD fed pregnant C57BL/6J mice	5% w/w in the diet for 14 weeks	Reduced weight gain, triglyceride, and total cholesterol level, glucose, and insulin in GTT	Li et al. (2013)
HFD and STZ-mediated T2DM Sprague–Dawley rats	500 mg/kg/day for 6 weeks	Reduced serum TC and LDL, β-cell apoptosis, fasting glucose, and insulin, HOMA-IR	Hu et al. (2018)
HFD-mediated T2DM mice models	400 mg/kg twice/day (800 mg/kg) for 10 consecutive weeks	Reduced weight gain, plasma cholesterol, triglyceride, LDL, VLDL, glucagon overexpression, and AUC in ITT	Khan and Jena (2016)
Healthy lean males	4 g/day for 4 weeks	Improved peripheral insulin sensitivity (from 71 ± 10 to 82 ± 16 μmol/kg min)	Kec et al. (2018)

Note: HFD: high-fat diet, T2DM: type 2 diabetes, BW: body weight, AUC: area under the curve, GTT: glucose tolerance test, HOMA-IR: homeostatic model assessment of insulin resistance, FBG-fasting blood glucose, ITT: insulin tolerance test, p-AMPK: phosphorylated-adenosine 5’- monophosphate-activated protein kinase, GLUT4: glucose transporter 4, STZ: streptozotocin, TC: total cholesterol, LDL: low-density lipoprotein, VLDL: very low-density lipoprotein.

#### 4.2.3 Ginsenosides

**FIGURE 6:**
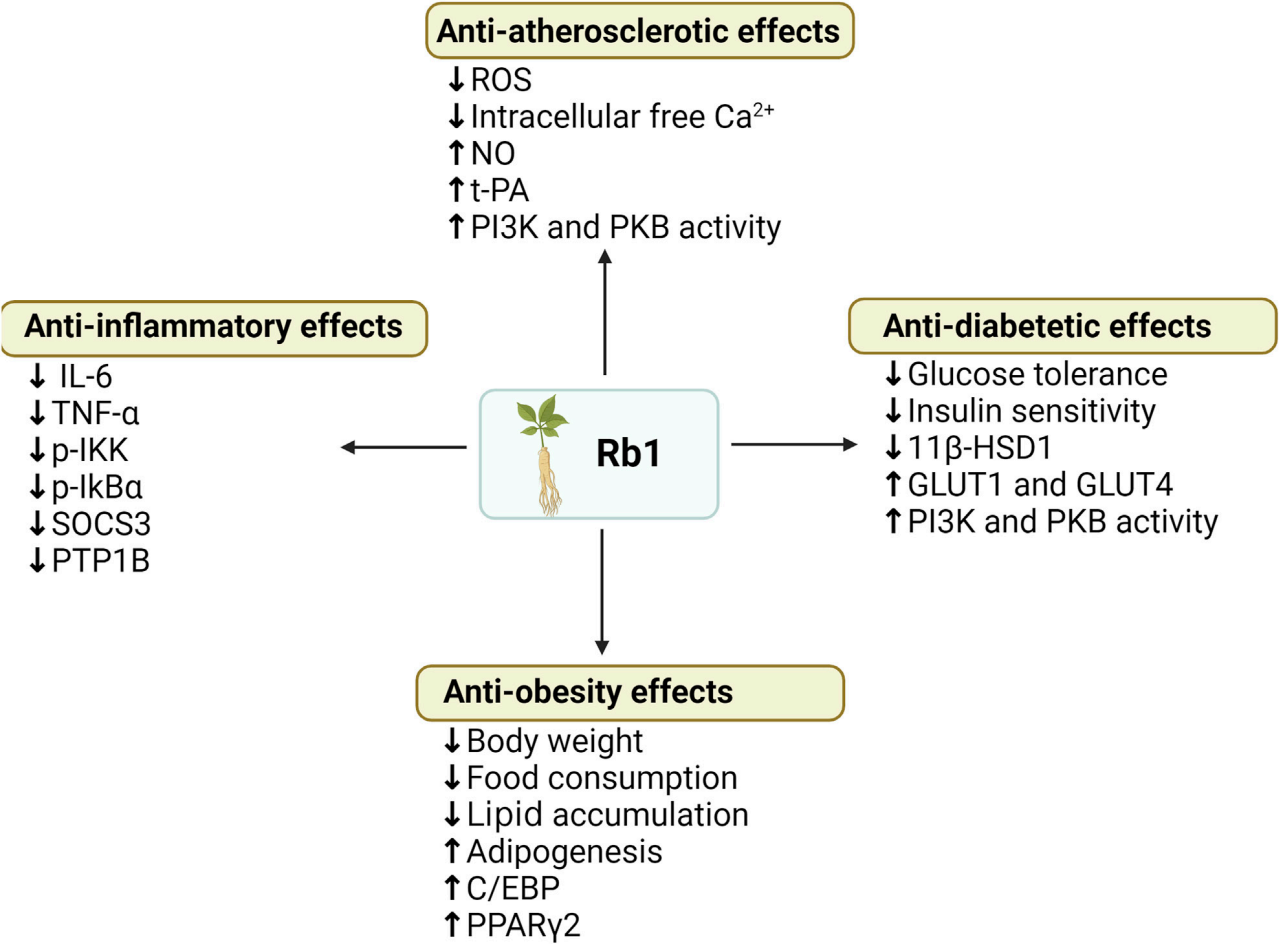
Summary of anti-obesity, anti-hyperglycemia, anti-atherosclerotic and anti-diabetic effects of ginsenoside Rb1. (↓), downregulation or inhibition; (↑) upregulation or activation.

Long-term use of statins and metformin in the treatment of MetS may cause side effects such as headache and dizziness. Therefore, it is urgent to discover new therapeutic targets for MetS and then reduce the side effects. Due to Chinese herbs’ characteristic low side effects, ginsenosides have been used in recent years to study the treatment of MetS. Ginsenosides are active ingredients in ginseng, which have rich pharmacological activities and different phenotypes in MetS. It regulates immune response, diabetes, and cardiovascular disease treatment (Shi et al., 2019). There are more than 100 different types of ginsenosides, of which Rb1, Rb2, Rc, Rd, Re, Rg1, and Rg3 are the most abundant, accounting for more than 90% of the total saponins in ginseng (Mohanan et al., 2018). Among them, ginsenoside Rb1 has been found to aid in the treatment of diabetes, obesity, and CVD and delay the development and progression of complications of these diseases by exerting insulin sensitization, anti-diabetic, anti-hyperglycemic, and anti-obesity properties, as shown in Figure 6. Ginsenoside Rb1 may exert these properties mainly by improving glucose tolerance, increasing insulin sensitivity, regulating adipocyte development and function, reducing liver fat accumulation, and inhibiting adipocyte lipolysis.

Current *in vivo* results (Table 4) show that intraperitoneal injection of Rb1 (10 mg/kg body mass, daily) in HFD-induced obese rats significantly reduced weight gain, body fat content, fasting blood glucose, and fasting plasma insulin, and improved glucose tolerance in HFD-induced obese rats compared with controls (Xiong et al., 2010). In HFD-induced T2DM mouse models, ginsenoside Rb1 (10 mg/kg body mass daily) after 21 days of treatment improved HFD-induced weight gain, fat mass accumulation, and glucose tolerance compared with the vehicle treatment group (Wu et al., 2014). Significantly, Rb1 treatment also reduced levels of pro-inflammatory cytokines (TNF-a and IL-6) in adipose tissue and the liver, which further inhibits the NF-kB pathway molecules (p-IKK and p-IkBα) and the leptin p-STAT3 signaling pathway, and reduces transcription of negative regulators of the low leptin signaling pathway (SOCS3 and PTP1B) (Wu et al., 2014). These studies suggest that Rb1 has the potential to be used in the treatment of obesity and diabetes, improving body weight and reducing obesity-induced inflammation, thereby improving insulin sensitivity. In addition, ginsenoside Rb1 treatment of HFD-induced T2DM mice improved fasting blood glucose, glucose tolerance, and insulin sensitivity, and GRb1 appeared to reduce levels of 11β-hydroxysteroid dehydrogenase type I (11β-HSD1) and its mRNA in adipose tissue and liver, to play its anti-diabetic role (Song et al., 2017). Moreover, after 14 days of intraperitoneal injection of ginsenoside Rb1 (20 mg/kg, daily) in db/db obese diabetic mice, homeostasis model assessment insulin resistance (HOMA-IR), fasting glucose, and fasting insulin were significantly reduced. Glucose consumption was increased in 3T3-L1 adipocytes of mice, GLUT1 and GLUT4 expression was upregulated, and AKT phosphorylation was restored in mouse adipose tissue (Shang et al., 2014). Therefore, ginsenoside Rb1 can activate the insulin signaling pathway (PI-3K-Akt), upregulate the expression of GLUTs in adipose tissue, and increase glucose uptake by adipose cells, thereby regulating glucose metabolism and insulin-sensitizing activity. *In vitro*, studies have also shown that ginsenoside Rb1 regulates adipose tissue metabolism, regulates glucose metabolism, and has insulin-sensitizing activity, among which mouse adipose cell 3T3-L1 cells have been widely used in studies (Hanél Sadie-Van, 2019).

**TABLE 4:** Summarized effects and mechanisms of ginsenoside Rb1 related to diabetes mellitus, obesity, and cardiovascular diseases *in vivo* and *in vitro* studies.

Study type	Models	Effect	Mechanism	References
*In vivo*	HFD-induced obese and T2DM male Long-Evans rats	↓ Food intake, weight gain, body fat content, fasting blood glucose, and fasting plasma insulin ↑ Energy expenditure	↑ c-Fos expression ↓ Neuropeptide Y and PI3k/Akt signaling pathway	Xiong et al. (2010)
*In vivo*	HFD-induced obese and T2DM C57Bl/6 male mice	↓ Body weight gain and fat mass accumulation ↑ Glucose tolerance	↑ Central leptin sensitivity ↓ Proinflammatory factor	Wu et al. (2014)
*In vivo*	HFD-induced T2DM C57BL/C female mice	↓ Fasting blood glucose and 11β-HSD1 levels ↑ Glucose tolerance	↑ Insulin sensitivity ↓11β-HSD1	Song et al. (2017)
*In vivo*	Male db/db mice	↓ HOMA-IR, fasting glucose, and fasting insulin ↑ GLUT1 and GLUT4, Akt phosphorylation	↑ Glucose uptake, glucose metabolism, and insulin-sensitizing activity	Shang et al. (2014)
*In vitro*	Differentiation inducer-induced 3T3-L1 adipocytes; C2C12 myotubes	↑ IRS1 and PKB phosphorylation, PI3K activity, and glucose uptake	↑ Partial the insulin signaling pathway	Shang et al. (2008)
*In vitro*	Differentiation inducer-induced 3T3-L1 cells	↓ The proliferation of pre-confluent 3T3-L1 preadipocytes ↑ Lipid accumulation and glucose uptake	↑ GLUT4, PPARγ2, C/EBPα, ap2 and adipogenesis	Shang et al. (2007)
*In vitro*	L-NAME; SH-5; PD98059; nilutamide	↑ eNOS and eNOS phosphorylation (Ser1177)	↑ PI3kinase/Akt and MEK/ERK pathway signals, and androgen receptor	Yu et al. (2007)
*In vitro*	oxLDL-injuring HUVECs	↓ PAI-1 ↑ NO and t-PA	↓ oxLDL from damaging human endothelial cells; oxLDL’s effects on NO, t-PA, and PAI-1	He et al. (2007)
*In vivo*	MCT-induce right ventricular hypertrophy rats	↓ CaN, NFAT3, and GATA4	↓ CaN signal transduction pathway	Jiang et al. (2007)

Note: HFD: high-fat diet, 11β-HSD1: 11β-hydroxysteroid dehydrogenase type I, HOMA-IR: homeostasis model assessment insulin resistance, GLUT: glucose transporter, Akt/PKB: kinase B, FINS: fasting insulin, PI3K: phosphatidylinositol 3-kinase, C/EBP: CCAAT/enhancer binding protein, PPARγ: Peroxisome proliferator-activated receptor γ, oxLDL: oxidized low-density lipoprotein, HUVECs: Human umbilical vein endothelial cells, t-PA: tissue-type plasminogen activator, PAI-1: plasminogen activator inhibitor-1, CaN: calcineurin, NFAT: nuclear factor of activated T cells, GATA4: GATA, binding factor-4.

*In vitro* studies on 3T3-L1 adipocytes and C2C12 myotubes demonstrated that ginsenoside Rb1 promotes translocation of GLUT1 and GLUT4 to the cell surface by activating phosphorylation of insulin receptor substrate-1 and PKB, thereby stimulating glucose transport in insulin-sensitive cells, and stimulated PI3K activity in the absence of the insulin receptor activation, which exerts hypoglycemic and anti-diabetic properties (Shang et al., 2008). Moreover, ginsenoside Rb1 promoted adipogenesis in 3T3-L1 preadipocytes dose-dependently, and lipid accumulation increased by about 56% after 10 μM Rb1 treatment. It is thought that ginsenoside Rb1 is partially involved in enhancing the expression of Peroxisome proliferator-activated receptor γ 2 (PPARγ2) and CCAAT/enhancer binding protein (C/EBP), thereby playing roles in anti-diabetic and insulin-sensitizing activities (Ambele et al., 2020). PPARγ2 and C/EBP are molecules that are recognized to regulate the differentiation process of preadipocytes into mature adipocytes and promote downstream adipose-specific gene expression, thereby exerting an influence on adipocyte abundance and promote reverse cholesterol transport and glucose and lipid metabolism (Rosen, 2005). It was also found that Rb1 significantly increased 3T3-L1 cellular glucose uptake and GLUT4 expression and promoted adipogenesis by increasing the expression of PPARγ2 and C/EBP, as well as ap2, one of their target genes, thereby exerting anti-diabetic, anti-obesity, and insulin-sensitizing activities (Shang et al., 2007).

In addition, ginsenoside Rb1 plays a vital role in the treatment of CVD due to its control of NO production, endothelial oxide synthetase (eNOS) expression, and Ca^2+^ channels. *In vitro*, it was found that Rb1 rapidly phosphorylates endothelial oxide synthetase (eNOS) (Ser1177) through I3 kinase/Akt and MEK/ERK pathways and androgen receptors, upregulates eNOS, thereby stimulating the production of NO in aortic endothelial cells (Yu et al., 2007). Another study showed that Rb1 could improve the effect of oxidized low-density lipoprotein (oxLDL) on NO production in oxLDL-injuring human umbilical vein endothelial cells (HUVECs) and reverse oxLDL’s effects on NO, tissue-type plasminogen activator (t-PA), and plasminogen activator inhibitor-1 (PAI-1) in endothelial cells (He et al., 2007). Compared with the control group, high-dose Rb1 (10 mg/mL) treatment blocked the effect of oxLDL on lactate dehydrogenase (LDH) activity and the levels of NO and t-PA, which are vascular endothelial active substances with various anti-atherosclerotic effects, were significantly increased, and the levels of PAI-1 were decreased (He et al., 2007). Rb1 also plays a role in ion channel regulation. Studies have found that Rb1 promotes NO release in cardiomyocytes, reduces intracellular free Ca^2+^, and reduces the expression of monocrotaline (MCT) -mediated transcription factors GATA binding factor-4 (GATA4) and nuclear factor of activated T cells 3 (NFAT3) by attenuating the calcineurin signal transduction pathway, thus playing an antihypertrophic effect (Jiang et al., 2007). Furthermore, ginsenoside Rb1 inhibits eating desire by regulating serum PYY during HFD-induced obesity (Lin et al., 2014).

Other ginsenosides also have anti-diabetic, anti-hyperglycemic, and anti-obesity effects. Previous studies have shown that ginsenoside Rb2 can effectively improve insulin sensitivity (Hong et al., 2019). In addition, it inhibited the increase of plasma triglyceride levels in diet-induced obese mice and prevented triglyceride deposition (Gu et al., 2013). Ginsenoside Rg1 reduces intestinal glucose uptake mediated by sodium-dependent glucose transporter 1, thereby lowering blood glucose and alleviating insulin resistance (Wang C. W. et al., 2015). Furthermore, the glycosyl moiety of ginsenoside Rg3 stimulates GLP-1 secretion in the intestine through a sweet taste receptor-mediated signal transduction pathway, thereby having an anti-hyperglycemic effect. Meanwhile, ginsenosides Rg1 and Rb3 could protect renal endothelial function and reduce hypertension by stimulating endothelial-dependent vessel dilatation and alleviating oxidative stress in renal arteries, respectively (Pan et al., 2012; Wang et al., 2014). The study revealed that ginsenoside Rc significantly reduced the body weight of obese mice induced by a high-fat diet. This effect was attributed to the upregulation of PPARα and liver glycogenic genes PPARγ coactivator l alpha (PGC-1α) in skeletal muscle by Rc, as well as the activation of the sirtuin 6/AMP-activated protein kinase (SIRT6/AMPK) pathway, thereby promoting the oxidative decomposition of fatty acids. Another study found that ginsenosides Rc significantly reduced insulin levels and fasting blood glucose in mice fed a high-fat diet, which may be related to RC-mediated downregulation of PEPCK, PGC-1a, and G6Pase expression (Yang et al., 2023). In addition to the above therapeutic effects, ginsenoside also has anti-ischemic heart disease, anti-atherosclerosis, and other effects (Fan et al., 2020). Therefore, ginsenosides have great potential in preventing and treating diabetes, obesity, and MetS by inhibiting adipogenesis, regulating insulin resistance, and lowering blood pressure.

#### 4.2.4 Hibiscus

Originating in Africa, hibiscus is a species of the Malvaceae family that includes more than 250 species of herbs, shrubs, and trees. The leaves, flowers, calyx, and bark of hibiscus are used as medicine due to their antioxidant properties, which destroy free radicals to reduce the risk of inflammatory maladies such as MetS and CVD (Jeffery and Richardson, 2021). The antioxidant properties of hibiscus are the fact that it contains saponins, alkaloids, terpenes, and a large quantity of polyphenols. A study in patients with MetS and spontaneously hypertensive rats found that polyphenols rich in *Hibiscus sabdariffa* (*H. sabdariffa*) calices reduced hypertension and TNF-α expression and increased eNOS and NO in endothelial cells (*p* < 0.05). Therefore, hibiscus plays an anti-inflammatory and antihypertensive role and should be a candidate for managing metabolic cardiovascular risks (Joven et al., 2014).

In addition to its anti-inflammatory capacity, hibiscus has also been identified to have pharmacological anti-diabetic and hyperglycemic properties for regulating insulin resistance and lowering blood glucose (Table 5) (Zahid et al., 2014; Amaya-Cruz et al., 2019). After intra-gastric administration of *Hibiscus rosa sinensis* (*H. Rosa-sinensis*), streptozotocin (STZ)-induced diabetic rats’ blood glucose and glycated hemoglobin (HbA1c), which can reflect chronic blood glucose, are significantly reduced. This suggests that *Hibiscus rosa-sinensis* controls long-term hyperglycemia (Pillai and Mini, 2016b). Moreover, compared with the control group, the mRNA expressions of NF-κB and P38MAPK were significantly downregulated after *H. rosa-sinensis* treatment, and the activities of antioxidant enzymes such as CAT, SOD, GPx, and GRd were increased considerably (Pillai and Mini, 2016b). Thus, *H. rosa-sinensis* may play an antidiabetic role by alleviating excessive ROS-mediated islet beta cell dysfunction. The study found that the overall effect of 25 mg/kg body weight *H. rosa-sinensis* treatment was comparable to metformin. Other studies also have compared hibiscus with anti-hyperglycemic medicines already on the market. Taking lovastatin, metformin, and glibenclamide as an example, a dose of 200 mg/kg *H. sabdariffa* had the same therapeutic effect as lovastatin in lowering blood glucose (Farombi and Ige, 2007). The therapeutic effect of 25 mg/kg flower extract from *H. rosa-sinensis* on insulin resistance was comparable to a 4-fold dose of metformin (Pillai and Mini, 2016a). The root extract from *H. rosa-sinensis* was as effective as glibenclamide in lowering fasting blood glucose (Aba et al., 2014). A human study found that patients with MetS who took hibiscus alone or combined with a preventive treatment diet for a month had lower blood glucose levels than pre-treatment (Gurrola-Díaz et al., 2010).

**TABLE 5:** Experimental models of the effect of hibiscus on blood glucose.

Hibiscus species	Plant part	Models	Dose	Effect	References
*Hibiscus sabdariffa*	Flower	Alloxan-treated rats	100 mg/kg/d	↓ Blood glucose, MDA, and PC ↑ CAT, SOD, and GSH	Huang et al. (2009)
*Hibiscus sabdariffa*	Calyx	Type 2 diabetic rat	100 mg/kg 200 mg/kg	↓ Blood glucose, insulin, and AGE	Peng et al. (2011)
*Hibiscus tiliaceus*	Flower	STZ-induced diabetic wistar rats	250 mg/kg/d 500 mg/kg/d	↓ Blood glucose	Kumar et al. (2014)
*Hibiscus rosa sinensis*	Root	Alloxan-induced diabetic rats	500 mg/kg/d	↓ Blood glucose	Kumar et al. (2013)
*Hibiscus rosa sinensis*	Flower	STZ-induced diabetic rats	25 mg/kg	↓ Blood glucose, HbA1c, AKT, PI3K and Nrf2 ↑ CAT, SOD, GPx, and GRd activities	Pillai and Mini (2016b)
*Hibiscus sabdariffa*	Calyx	MetS and non-MetS individuals	100 mg/d	↓ Blood glucose, TAG/HDL-c ratio	Gurrola-Díaz et al. (2010)

Note: MDA: malondialdehyde, PC: protein carbonyl, CAT: catalase, GSH: glutathione, SOD: superoxide dismutase, AGE: advanced glycation end product, STZ: streptozotocin, HbA1c: glycated hemoglobin, PI3K: phosphatidylinositol 3-kinase, GPx: glutathione peroxidase, GRd: glutathione reductase, TAG/HDL-c: triglycerides/high-density lipoprotein cholesterol ratio.

In addition to the anti-hyperglycemic effect, hibiscus has anti-lipidemic and anti-atherosclerotic activities. Multiple studies have shown that when animals have a high-fat diet, hibiscus generally inhibits increases in LDL cholesterol and TG and sometimes increases HDL cholesterol, and the therapeutic effect is dose-dependent (Lee et al., 2018) (Table 6). A study on the effects of ethyl acetate, an extract of the flower *H. rosa sinensis* flowers (HRF), on 3T3-L1 cells found that HRF reduced triglyceride content and intracellular lipid accumulation by activating AMPK (Lingesh et al., 2019). After activation of AMPK, phosphorylated AMPK inhibited the expression of cholesterol synthesis rate-limiting enzyme HMGCR and sterol-regulatory element binding protein 1c (SREGBP-1C), indirectly decreased the downstream expression of fatty acid synthase (FAS) and acetyl-CoA carboxylase (ACC), and decreased TG levels (Lingesh et al., 2019). Or directly inhibit ACC activity, decrease ACC expression, reduce the inhibition of carnitine palmitoyl transferase 1 (CPT-1), a key enzyme for fatty acid β-oxidation, promote fatty acid oxidation, and reduce TG synthesis (Lingesh et al., 2019). Another study found that *H. sabdariffa* calices also significantly reduced TG, total cholesterol, and LDL levels in obese adolescents. Therefore, *H. sabdariffa* is expected to prevent and treat dyslipidemia and atherosclerotic diseases in adolescents (Sabzghabaee et al., 2013). After consuming 5%, 10%, and 15% of *H. sabdariffa* extract in high-calorie diet-mediated rats for 28 days, the TG and LDL levels of the three groups of mice were reduced (Farombi and Ige, 2007). The total cholesterol level of rats treated with the 5% *H. sabdariffa* extract group was significantly decreased compared with the 10% and 15% *H. sabdariffa* extract groups (Farombi and Ige, 2007). Therefore, 5% extract has the best effect on reducing blood lipids in rats, and the effect of H. sabdariffa extract on treating dyslipidemia is dose-dependent. Moreover, *in vitro* studies of high-glucose treated vascular smooth muscle cells (VSMC) found that polyphenolic isolate of *H. sabdariffa* (HPI) timing (24 h–96 h) and dose (HPI, 0.01 mg–1.0 mg) dependently reduces the proliferation and migration of high-glucose stimulated cells by inhibiting the activity of matrix metalloproteinase (MMP), an extracellular matrix (ECM) degrading enzyme during cell migration (Huang et al., 2009). HPI may, therefore, be a promising herb for treating atherosclerosis caused by the proliferation and migration of VSMCs.

**TABLE 6:** Experimental models of the effect of hibiscus in dyslipemia.

Hibiscus species	Plant part	Models	Dose	Effect	Mechanism	References
*Hibiscus rosa sinensis*	Flower	Differentiation inducer-induced 3T3-L1 adipocytes	25 μg/mL 50 μg/mL	↓ PPAR-γ, C/EBPα, SREBP-1C, FABP4, FAS, Perilipin and adiponectin, ACC ↑ AMPK	↓ Triglyceride accumulation ↑ CPT-1, fatty acid β-oxidation	Lingesh et al. (2019)
*Hibiscus sabdariffa*	Calyx	Obese adolescents	6 g/d	↓ Serum total cholesterol, LDL, and TG	↑ Anti-lipidemic and antioxidant activities	Sabzghabaee et al. (2013)
*Hibiscus sabdariffa*	Calyx	High-caloric diet-induced rats	5, 10, and 15 g of extract/100 g diet	↓ Serum total cholesterol, LDL, and TG	↓ Triacylglycerol synthesis	Farombi and Ige (2007)
*Hibiscus sabdariffa*	Not stated	New Zealand White rabbits	0.5% and 1% diet	↓ Serum total cholesterol, LDL, and TG	↑ Anti-lipidemic activities	Chen et al. (2003)
*Hibiscus sabdariffa*	Flower	Alloxan-treated rats	200 mg/kg/d	↓ Serum total cholesterol, VLDL, LDL, MDA, and PC ↑ SOD, CAT, and GSH	↑ Anti-lipidemic and antioxidant activities	Farombi and Ige (2007)
*Hibiscus rosa sinensis*	Flower	STZ-induced diabetic rats	250 mg/kg/d 500 mg/kg/d	↓ Serum total cholesterol, LDL, and TG	↑ Anti-lipidemic activities	Bhaskar (2011)
*Hibiscus rosa sinensis*	Flower	STZ-induced diabetic rats	250 mg/kg/d 500 mg/kg/d	↓ Serum LDL and TG ↑ HDL	↑ Anti-lipidemic activities	Bhaskar and Vidhya (2012)
*Hibiscus rosa sinensis*	Flower	MSG-induced obese albino rats	1,000 mg/kg/d	↓ Free fatty acids, TG, phospholipids, TC, VLDL, and LDL ↑ HDL	↑ Anti-lipidemic activities	Gomathi et al. (2008)

Note: PPARγ: Peroxisome proliferator-activated receptorγ, C/EBP: CCAAT/enhancer binding protein, FABP4: fatty acid binding protein-4, FAS: fatty acid synthase, ACC: acetyl-CoA, carboxylase, CPT-1: carnitine palmitoyl transferase-1, LDL: low-density lipoprotein, TG: triglyceride, VLDL: very low-density lipoprotein, MDA: malondialdehyde, PC: protein carbonyl, SOD: superoxide dismutase, CAT: catalase, GSH: glutathione, STZ: streptozotocin, MSG: monosodium glutamate.

### 4.3 Other treatments for metabolic syndrome

#### 4.3.1 Mediterranean diet

The Mediterranean diet (MedDiet) refers to the dietary patterns and cooking methods followed by people living in the Mediterranean Basin (Barreto et al., 2014). MedDiet calls for daily intake of whole grains, low-fat dairy products, vegetables, fruits, and legumes; monthly consumption of red meat; moderate intake of fish, red wine, eggs, and poultry; olive oil as the primary source of lipids; regular physical activity (Bagetta et al., 2020). Compounds that are beneficial to human health are found in foods advocated in the MedDiet, such as fruits, nuts, vegetables, grains, red wine, and olives, including polyphenols, polyunsaturated fatty acids (PUFAs), monounsaturated fatty acids (MUFAs) and anti-oxidants (vitamin A, C, and E) which have been linked to lower TG levels, reduced risk of CVD, a stable glycemic index (GI) and lower LDL oxidation (Bagetta et al., 2020). In the MedDiet, fruits and vegetables are the primary sources of polyphenols; polyphenols have sound antioxidant effects, can block oxidative stress, reduce the plasma concentration of LDL, increase the plasma concentration of HDL, and thus improve IR, dyslipidemia, and hypertension (Liu et al., 2019). Resveratrol is also the primary polyphenol in red wine. It can activate sirtuin 1, which is involved in lipolysis, and adenosine monophosphate protein kinase, increasing insulin sensitivity (Hou et al., 2019). Besides, it also has anti-inflammatory effects. Polyphenols contained in olive oil may reduce the risk of developing MetS by lowering blood pressure, IR, lipid peroxidation, and visceral obesity, while polyphenols also block the signaling pathways of NF-κB and reduce pro-inflammatory cytokines secretion (Priscilla et al., 2017). In a 6-week clinical trial, randomized, 8 g dried grape residue (mainly containing polyphenols such as proanthocyanidins) or placebo was given daily to 50 subjects exhibiting two or more MetS abnormal medical phenotypes. After 6 weeks, the subjects’ quantitative insulin sensitivity test index (QUICKI) elevated from 0.42, HOMA-IR reduced significantly from 2.1 to 1.4, and insulin resistance improved (Hui et al., 2018). In another 12-week clinical trial, 33 subjects with central obesity who had a body mass index (BMI) of more than 25 kg/m^2^ or a waist circumference of more than 80 cm (female) or 94 cm (male) were randomly distributed into two groups. 500 g/day of Caralluma Fimbriata extract (gallic acid) and placebos were administered. After exercise and diet intervention, body weight, BMI, body weight, total fat, triglyceride levels, and saturated fat intake were significantly reduced in both groups, but the decline was more pronounced in the C. fimbriata extract group (Astell et al., 2013). Moreover, olive oil also contains MUFA, such as oleic acid, which inhibits angiotensin-converting enzyme, thereby regulating blood pressure, as well as increasing HDL concentration, reducing plasma LDL concentration, and improving dyslipidemia (Massaro et al., 2020). Antioxidants such as vitamins A, C, and E, which are contained in the MediDiet, may increase insulin secretion, promote weight loss, and alleviate IR (Said et al., 2021). In addition, these vitamins reduce reactive oxygen species and pro-inflammatory cytokine production, improve lipid metabolism, and protect cardiovascular health (Villa-Etchegoyen et al., 2019). Moreover, MedDiet includes fruits and vegetables with high amounts of fiber, which helps with weight control and provides a sense of satiety.

The dietary components of MedDiet, which have antioxidant and anti-inflammatory properties, reduce the incidence of CVD and T2DM and also reduce the severity and associated complications in established patients, which may be a possible explanation for the relief of MetS by MedDiet. Substantial scientific evidence suggests high adherence to this dietary pattern can prevent and delay many diseases, such as T2DM, CVD, Alzheimer’s disease, and MetS (Stadlbauer et al., 2015). Table 7 summarizes studies on the link between MedDiet and MetS.

**TABLE 7:** Studies on the effects of the Mediterranean diet pattern on metabolic syndrome.

Study type	Studied model and method	Result	References
Post hoc analysis of the CARDIA study	4,713 Americans participated in the Coronary Artery Risk Development in Young Adults study. To evaluate the association of modified MedDiet with the incidence of MetS over 25 years	When young American adults consume dietary patterns rich in whole grains, vegetables, fruits, nuts, and fish, they have a lower risk of MetS	Steffen et al. (2014)
RCT Post hoc analysis of the LIPGENE study	472 Europeans with MetS were randomly assigned to one of four diets: an HSFA diet, a HMUFA diet, a LFHCC diet supplemented with LFHCC n-3 (1.2 g/d), or an LFHCC diet supplemented with placebo (control) for 12 weeks	MetS subjects with IR and more metabolic complications responded differently to dietary fat changes. They were more susceptible to the health effects of replacing the SFAs in the high-monounsaturated fatty acid and low-fat, high-complex carbohydrate diets. Conversely, MetS subjects without IR may be more sensitive to the harmful effects of high-saturated fatty acid intake	Elena et al. (2015)
RCT Post hoc analysis of SU.VI.MAX study	In 3,232 French, the association between MedDiet and 6-year MetS risk was assessed	Adherence to the MedDiet was associated with a lower risk of MetS	Kesse-Guyot et al. (2013)
Post hoc analysis of TLGS study	A robust food frequency questionnaire was used to collect dietary data from 425 Iranians aged 6–18. The 95% confidence interval for assessing the occurrence of MetS after 3.6 years on different DASH-style diets	In children and adolescents, adherence to the DASH is associated with a lower incidence of MetS	Golaleh et al. (2016)
RCT Post hoc analysis of the LIPGENE study	75 Europeans with MetS were randomly assigned to one of four diets: HSFA, HMUFA, and LFHCC diets, supplemented with LFHCC n-3 or placebo for 12 weeks	Long-term consumption of a healthy dietary model containing HMUFA can reduce postprandial inflammatory states associated with MetS	Cruz-Teno et al. (2012)

Note: HSFA: high saturated fatty acids, HMUFA: high monounsaturated fatty acids two low-fat, LFHCC: low-fat, high complex carbohydrate, LFHCC n-3: long-chain n-3 polyunsaturated fatty acids, SFAs: saturated fatty acid.

#### 4.3.2 High-protein diet

A high-protein diet refers to foods that contain more protein in the daily diet. 20%–30% of the daily energy intake in a high-protein diet comes from protein (Stadlbauer et al., 2015). The International Nutrition Society recommends a standard-protein diet of 0.8 g grams of protein per kilogram of body weight for healthy adults. In contrast, the high-protein diet recommends that people consume 1.34–1.5 g of protein per kilogram of body weight per day. At present, studies have found that a high-protein diet may improve glucose homeostasis, lower lipids, lower blood pressure, and lower body mass (Layman et al., 2009; Pedersen et al., 2013; Daly et al., 2014; Heer and Egert, 2015), so it has been proposed to control blood glucose and treat obesity and MetS. However, controlling blood glucose and weight on high-protein diets remains uncertain and controversial. A meta-analysis of 18 randomized controlled trials involving 1,099 adults with T2DM showed that the high-protein diet group had no significant weight and blood pressure reduction compared to low-protein diets but triglyceride levels reduction (Zhao et al., 2018). However, after 6 months on a standard protein diet and a high-protein diet in a randomized group of 118 adults with MetS, overall weight loss was 5.1 ± 3.6 kg in the SPD group and 7.0 ± 3.7 kg in the high-protein diet group (Campos-Nonato et al., 2017). In another study, obese adults with MetS lost 7.4% (95% CI 5.7%–9.2%) of their body weight after adhering to a high-protein diet that limited cholesterol (Rock et al., 2014). Moreover, in another study, 100 overweight and obese older adults (55–80 years old) were randomly assigned to a standard or high-protein diet and to do or not do resistance exercise during a 10-week weight loss period. Measure the change in fat-free mass (FFM). Analysis of in-group results showed that high protein binding exercise significantly increased FFM (+0.6 ± 1). 3 kg, *p* = 0.011) (Verreijen et al., 2015). Therefore, adherence to a high-protein diet is effective for weight loss in obese patients with MetS, with even more weight loss combined with exercise. Some studies have also found that high protein intake increases feelings of fullness and reduces energy intake at the next meal (Halton and Hu, 2004; Eisenstein et al., 2010). The high protein intake helps to increase the thermal effect of eating and maintain rest energy expenditure during energy restriction, thus promoting weight loss.

In a randomized, controlled, cross-feeding, three-phase study, participants were 164 patients with high blood pressure and no diabetes. The patients received three dietary interventions: a high-carbohydrate diet, a high-protein diet, and an unsaturated fat diet. Changes in the QUICKI, an adequate measure of insulin sensitivity, were then assessed. Each diet lasts 6 weeks, with a 2 to 4-week washout between diets (Gadgil et al., 2013). The results showed that the insulin sensitivity was not improved significantly with a high-protein or high-cholesterol dietary pattern, whereas it was improved with an unsaturated diet, which suggests that replacing a high-protein or high-cholesterol dietary with an unsaturated fat diet (e.g., MedDiet) is one way to improve insulin sensitivity (Gadgil et al., 2013). Subsequent studies, however, have shown different results. In a 21-day randomized trial, 16 morbidly obese women with insulin resistance were divided into two groups and given an equal-calorie Mediterranean diet or a high-protein diet intervention. The results showed that the high-protein diet was more effective in reducing insulin resistance (HOMA-IR: −1.78 [95% CI: 3.03−−0.52]) and regulating blood glucose steady state (−3.13 [−4.60, −1.67] mg/dL) (Menni et al., 2021). In the future, more experiments are needed to demonstrate the effect of a high-protein diet on insulin sensitivity. Table 8 summarizes studies on the link between the high-protein diet and MetS.

**TABLE 8:** Studies on the effects of high protein diet pattern on metabolic syndrome.

Study type	Models	Method	Result	References
Meta-analysis	1,099 adults with T2DM	Participants with T2DM were applied and compared on HP and LP diets	Compared to the LP diet, the HP diet had no significant effect on blood pressure in people with type 2 diabetes. However, overall analysis showed that the HP diet significantly impacts the reduction of triglycerides	Zhao et al. (2018)
Randomized controlled trial	100 overweight and obese adults (55–80 years)	2 × 2 factor design and intention-to-treat analysis were used. During the 10-week weight loss program, participants were randomly assigned to a normal protein diet (0.8 g/kg body weight) or a high-protein diet (1.3 g/kg body weight) three times per week with or without resistance exercise	Within-group analysis showed that exercise in combination with high protein significantly increased fat-free mass	Verreijen et al. (2017)
Randomized controlled trial	20 insulin-resistant obese women	Participants were randomly assigned to one of the following dietary sequences: the Mediterranean-HP diet (n = 6) or the HP-Mediterranean diet (n = 11)	Compared with the Mediterranean diet, the HP diet more effectively reduced insulin resistance; Improved HOMA-IR and blood glucose variability	Tettamanzi et al. (2021)
Randomized controlled trial	118 adults aged 47.4 ± 11.5 years with MetS	Participants were randomly assigned to a prescribed low-calorie diet that provided either the SP diet (0.8 g/kg body weight) or the HP diet (1.34 g/kg body weight) for 6 months	Participants taking the HP diet had a higher weight loss percentage than those taking the SP diet. There was a significant interaction between mean weight loss and adherence	Campos-Nonato et al. (2017)
Randomized controlled trial	40–56 years Participants	After short-term weight loss (4 months) and weight maintenance (8 months), participants were put on a moderate PRO (protein: 1.6 g/kg/d, <170 g/d carbohydrates) or CHO diet (protein: 0.8 g/kg/d, >220 g/d)	Using participants meeting the compliance criteria of 0.10% weight loss, more participants in the increased PRO group lost more weight and fat mass. Compared with CHO, increased PRO had sustained beneficial effects on serum TAG, HDL-c, and TAG: HDL-c at 4 and 12 months	Layman et al. (2009)

Note: LP: low protein, HP: high protein, PRO: protein and reduced carbohydrates, CHO: conventional high-carbohydrate, TAG: triglycerides, HDL-c: high-density lipoprotein cholesterol.

Foods containing primarily protein, such as meat and processed meats, are related to higher risks for T2DM, CVD, and MetS, so dietary guidelines recommend choosing protein-rich plant-based foods such as soy, legumes, and nuts over meat and processed meats in a high-protein diet (Stadlbauer et al., 2015). Moreover, the study suggests that weight loss and CVD improvement are more significant with a high-protein diet pattern than with a standard-protein diet (Stadlbauer et al., 2015).

Current research has found that a high-protein diet is expected to improve weight regain after weight loss (Zarrinpar, 2022). After short-term dietary restriction, weight gain caused by ingestion is due to increased intestinal lipid absorption, fatty acids and triglycerides synthesis, and white adipose tissue. Studies have found that this is related to the fact that a high-protein diet can reduce intestinal lipid absorption and improve the utilization of fatty acids (Zhong et al., 2022). Microbiome analysis of the mice’s gut showed a significant increase in a *Lactobacillus* murinus species (named Lam-1) in the mice’s cecum after the short-term dietary restriction in all refeeding scenarios, but Lam-1 was suppressed by a high-protein diet. When specific antibiotics inhibited Lam-1, refeed-induced adipose tissue accumulation after the short-term dietary intervention was inhibited. Lam-1 treatment of gnotobiotic mice increased fatty acid and intestinal lipid uptake in white adipose tissue. Lam-1 induced increased intestinal fatty acid absorption was associated with five specific metabolites: 4-hydroxyphenyl acetic acid (HPLA), 1-alcohol-lactic acid, 2-hydroxyisopropionic acid (HICA), 1-indole-lactic acid (ILA), 2-hydroxy-3-methylbutyric acid (HMBA), and DL-3-phenyllactic acid (PLA) (Zarrinpar, 2022). This is attributed to the impact of Lam-1 and its intestinal metabolites on suppressing fatty acid oxidation while enhancing lipid absorption in white adipose tissue (WAT), collectively contributing to refeed-induced obesity following short-term dietary intervention.

#### 4.3.3 Time-restricted eating

Circadian regulation of the autonomic nervous system, endocrine system, and nutrient metabolism contribute to physiological and metabolic homeostasis (Panda, 2016). Erratic eating patterns, such as prolonged periods of food intake per day and irregular mealtimes, can disrupt circadian rhythms. Chronic circadian rhythm disorders lead to high blood pressure, obesity, insulin resistance, inflammation, and dyslipidemia, which increase the risk of metabolic syndrome (Pot et al., 2016; Zarrinpar et al., 2016; Ha and Song, 2019). Therefore, novel approaches to modify circadian rhythms by restoring dietary patterns are promising for patients with MetS. Improving diet patterns and lifestyle is an essential measure for the treatment and intervention of MetS. Time-restricted eating (TRE) is one of the emerging dietary interventions in which patients eat at a fixed time but do not engage in any prohibited calorie restriction or dietary composition changes to sustain consistent daily fasting and feeding cycles to maintain a robust circadian rhythm (Świątkiewicz et al., 2021; Das and Webster, 2022). Since TRE does not require restricting food intake or changing diet patterns, this method of dietary control may be an easier way to maintain a healthy weight in the long term. It alleviates circadian rhythm disruption by introducing continuous fasting periods of 12–16 h per day without altering total food intake. This method imposes an eating-fasting cycle, which effectively controls the patient’s eating patterns, regulates the circadian rhythms, and improves the metabolic regulation mechanisms, thus benefiting the treatment of MetS. In diet-induced obesity (DIO) mice models, time-restricted feeding (TRF) restores metabolic regulators in circulation, such as CREB and AMPK, as well as circadian clock oscillation. Moreover, the body weight of TRF mice was significantly reduced, and glucolipid homeostasis was improved considerably, which was also accompanied by reduced inflammation and steatosis (Panda, 2016). In the ovariectomized (OVX) female mice models, TRF effectively reversed metabolic syndrome in mice by reducing body weight, insulin resistance, and circulating triglycerides and cholesterol (Chung et al., 2016). Several studies of TRE have focused on healthy people or people who are overweight but do not have metabolic disease. In overweight men, 9-h TRE improved insulin resistance and glycemic response to test meals (Hutchison et al., 2019a). In men with prediabetes, 6-h TRE relieves insulin resistance signs but does not reduce energy intake (Sutton et al., 2018a). These studies suggest that TRE can prevent MetS. In a study of patients with MetS, a reduction in participants’ daily eating window from greater than 14 h to a time-restricted diet of 10 h resulted in weight loss, lower levels of cardiovascular disease, and lower blood pressure. TRE is likely to act as an “add-on” therapy to antihypertensive medications (such as statins) in patients with MetS. Table 9 summarizes studies on the link between the TRE and MetS. However, TRE strictly controls the eating time, changes the nutrition intake, increases physical activity, and supports a low-calorie diet to address the disorder of metabolic homeostasis, leading to poor patient compliance. Large-scale clinical trials are still needed to confirm the effects of TRE in preventing and treating MetS. Furthermore, because this stage is challenging to maintain over a long period, its efficacy in decreasing cardiometabolic risk in MetS patients is limited.

**TABLE 9:** Studies on the effects of time-restricted eating (TRE) pattern on metabolic syndrome.

Study type	Models	Method	Result	References
Randomized control trial	60 obese participants aged 18 to 65	10 h TRE intervention for 8 weeks	↓ Body weight and fasting blood glucose	Peeke et al. (2021)
Randomized control trail	20 overweight participants (17 females, 3 males) aged 33 to 58	8 h TRE intervention for 12 weeks	↓ Body weight, lean mass, and visceral fat mass	Chow et al. (2020)
Longitudinal trail	10 overweight participants (6 females, 4 males) aged 65 years or older	8 h TRE intervention for 4 weeks	↓ Body weight ↑ Quality of life	Anton et al. (2019)
Longitudinal trail	8 overweight participants (3 females, 5 males) aged over 18	10–11 h TRE intervention for 16 weeks	↓ Body weight and improved sleeping	Gill and Panda (2015)
Longitudinal trail	82 normal-weight participants (64 females, 18 males) mean age 31	eTRE 8 h (6 a.m.–3 p.m.) vs mTRE 8 h (11 a.m.–8 p.m.) vs. Control for 5 weeks	Weight loss and improved HOMA-IR in the eTRE group	Xie et al. (2022)
Longitudinal trail	21 overweight participants (18 females, 3 males) aged 35–60	8 h TRE intervention (12 p.m.–8 p.m.) and exercise vs. exercise alone for 8 weeks	↓ Body weight ↑ Quality of life	Kotarsky et al. (2021)
Longitudinal trail	20 overweight and obese participants (17 females, 3 males) aged 18–65	12 h TRE intervention for 8 weeks	↑ Bone mineral content	Lobene et al. (2021)
Longitudinal trail	58 overweight participants (53 females, 5 males) aged over 18	TRE 4 h (3–7 p.m.) vs. TRE 6 h (1–7 p.m.) vs. Control for 10 weeks	↓ Body weight and insulin resistance in TRE groups, no difference 4 h vs. 6 h	Cienfuegos et al. (2020)
Cross-over trail	15 overweight male participants aged 52–58	Early TRE: 9 h (8 a.m.–5 p.m.) delayed TRE: 9 h (12 p.m.–9 p.m.) for 1 week	Body weight, fasting TG, and hunger ↓ Mean fasting glucose by CGM in eTRE ↑ Glucose tolerance	Hutchison et al. (2019b)
Cross-over trail	12 participants (10 females, 2 males) aged over 20	early dinner (6 p.m.) vs. late dinner (9 p.m.) for 3 days	↓ Mean 24 h glucose ↓ RQ after breakfast	Nakamura et al. (2021)
Cross-over trail	11 overweight male participants aged 32–43	TRE: 8 h (10 a.m.–6 p.m.) Extended eating: 15 h (7 a.m.–10 p.m.) for 5 days	↓ Night-time glucose, glucose, and insulin iAUC after lunch ↑ TG after lunch	Parr et al. (2020)
Cross-over trail	8 overweight male participants aged 47–65	TRE: 6 h (8 a.m.–2 p.m., dinner before 3 p.m.) for 5 weeks	↓ Fasting TG, desire to eat in the evening ↑ Insulin sensitivity, β cell responsiveness	Sutton et al. (2018b)

Note: eTRE: early TRF (eTRF, food intake restricted to the early part of the day), mTRF: mid-day TRF (mTRF, food intake restricted to the middle of the day), TG: triglyceride, CGM: continuous glucose monitoring.

## 5 Conclusion

MetS is a multifaceted pathophysiological condition primarily stemming from an imbalance in caloric intake and energy expenditure, yet it is also modulated by factors such as an individual’s genetic/epigenetic constitution and lifestyle behaviors. The pathogenesis of metabolic syndrome is mainly mediated by increased free fatty acids leading to insulin resistance and chronic low-grade inflammation induced by pro-inflammatory cytokines such as IL-6, TMF-α, and leptin. Additionally, fetuin-A, considered a potential fat factor, mediates inflammation and mitochondrial dysfunction-induced ROS imbalance, which is also the pathogenesis of MetS. The prevalence of MetS is increasing annually, and its treatability remains uncertain; however, ongoing research appears to be unraveling different targets on the disease pathway. Currently, the focus is on treating insulin resistance and chronic inflammation and associated with MetS, which may provide the most comprehensive success in achieving these goals. Traditional treatment of MetS relies on statins and metformin. To improve the safety and effectiveness of drug therapy, probiotics, butyric acid, ginsenosides, and hibiscus have now been identified as promising novel treatments for MetS. Additionally, a combination of dietary adjustments, including the Mediterranean diet, high-protein diet, and time-restricted eating, can also be helpful in the prevention and management of metabolic syndrome.

## 6 Limitations of existing studies and possible future improvements

MetS is a complex metabolic disorder involving multiple risk factors for cardiovascular diseases, the leading cause of death worldwide. Insulin resistance, chronic inflammation and oxidative stress participate in the pathogenesis of MetS, but unfortunately, the core mechanism is still not clear by now. As a result, the present treatment of MetS is still restricted to relieving the symptoms, including hyperglycemia, hyperlipidemia, and hypertension. The mixing of medicines undoubtedly increased the side effects and facilitated the damage to the kidneys and liver. The mitochondrion is the center of cell metabolism, where the final steps of the oxidation of carbohydrates, lipids, and proteins are complete through the tricarboxylic acid cycle, and it is also the primary source of ROS. Therefore, mitochondrial dysfunction might be one of the underlying mechanisms of MetS, and further study by targeting mitochondria would provide some new strategies for clinical practice.
